# Association Mapping Reveals Genetic Loci Associated with Important Agronomic Traits in *Lentinula edodes*, Shiitake Mushroom

**DOI:** 10.3389/fmicb.2017.00237

**Published:** 2017-02-17

**Authors:** Chuang Li, Wenbing Gong, Lin Zhang, Zhiquan Yang, Wenyan Nong, Yinbing Bian, Hoi-Shan Kwan, Man-Kit Cheung, Yang Xiao

**Affiliations:** ^1^Institute of Applied Mycology, Huazhong Agricultural UniversityHubei, China; ^2^Institute of Bast Fiber Crops, Chinese Academy of Agricultural SciencesChangsha, China; ^3^College of Informatics, Huazhong Agricultural UniversityHubei, China; ^4^School of Life Sciences, The Chinese University of Hong KongHong Kong, Hong Kong

**Keywords:** shiitake mushroom, microsatellite, InDel, quantitative trait, association analysis

## Abstract

Association mapping is a robust approach for the detection of quantitative trait loci (QTLs). Here, by genotyping 297 genome-wide molecular markers of 89 *Lentinula edodes* cultivars in China, the genetic diversity, population structure and genetic loci associated with 11 agronomic traits were examined. A total of 873 alleles were detected in the tested strains with a mean of 2.939 alleles per locus, and the Shannon's information index was 0.734. Population structure analysis revealed two robustly differentiated groups among the Chinese *L. edodes* cultivars (*F*_*ST*_ = 0.247). Using the mixed linear model, a total of 43 markers were detected to be significantly associated with four traits. The number of markers associated with traits ranged from 9 to 26, and the phenotypic variations explained by each marker varied from 12.07% to 31.32%. Apart from five previously reported markers, the remaining 38 markers were newly reported here. Twenty-one markers were identified as simultaneously linked to two to four traits, and five markers were associated with the same traits in cultivation tests performed in two consecutive years. The 43 traits-associated markers were related to 97 genes, and 24 of them were related to 10 traits-associated markers detected in both years or identified previously, 13 of which had a >2-fold expression change between the mycelium and primordium stages. Our study has provided candidate markers for marker-assisted selection (MAS) and useful clues for understanding the genetic architecture of agronomic traits in the shiitake mushroom.

## Introduction

Mushrooms have been consumed for their high nutritional and medicinal values for millennia. The production of mushrooms is environmental friendly, due to its capability of converting lignocellulosic waste materials into food, feed and fertilizers (Fan et al., 2006). Due to their importance in industry, agriculture, medicine, and health, the demand for mushrooms is huge and increasing rapidly worldwide (FAOSTAT, http://faostat.fao.org/).

*Lentinula edodes* (Berk.) Pegler, also called Xianggu or shiitake, is one of the most popular edible mushrooms worldwide. *L. edodes* was firstly cultivated at least 900–1,000 years ago in China, and is now the second most widely produced mushroom in the world after *Agaricus bisporus* (Miles and Chang, 2004). *L. edodes* is rich in minerals, vitamins, essential amino acids, and lentinan (Chang, 1980). It also has immunomodulatory (Xu et al., 2015), anticancer (Nagashima et al., 2013), and antiviral functions (Di Piero et al., 2010). Moreover, *L. edodes* could prevent environmental impacts caused by the accumulation of forest and agricultural wastes since it secretes hydrolytic and oxidative enzymes that are responsible for the degradation of organic substrates (Silva et al., 2005).

Breeding elite cultivars is important for sustainable development of the modern mushroom industry. The fruiting body of *L. edodes* is the main target of breeding schemes. Strains with a fast mycelium growth rate (MGR), high precocity, fine morphological characteristics of fruiting body, and high yield are selected as cultivars. The majority of important agronomic traits of *L. edodes* are quantitative traits controlled by multiple genes or quantitative trait loci (QTLs), which are highly influenced by the environment and show a continuous variation (Santoyo et al., 2008). Dissecting the genetic basis of important agronomic traits is essential for marker-assisted selection (MAS), which could be integrated into conventional breeding schemes in *L. edodes* and thus enhancing the breeding efficiency.

Linkage mapping was first introduced to understand the genetic basis of quantitative traits since the 1980s (Lander and Botstein, 1989). Since the 2000s, this approach has been successfully used to map QTLs controlling important traits in several mushroom species, such as *A. bisporus* (Foulongne-Oriol et al., 2012a,b), *Pleurotus ostreatus* (Larraya et al., 2002, 2003) and *P. eryngii* (Im et al., 2016). However, linkage mapping requires the construction of segregating populations, which is time-consuming and laborious, thus limiting its application in breeding programs.

As a complementary method of linkage mapping, association mapping based on linkage disequilibrium (LD) is rapidly becoming a powerful strategy for dissecting the genetic architecture of complex traits in plants (Ingvarsson and Street, 2011). Compared to linkage mapping, association mapping has the following advantages: (1) it employs naturally occurring variations in diverse germplasms; (2) it assesses a wide range of alleles rapidly; and (3) it possesses a higher mapping resolution (Yu and Buckler, 2006). Association mapping have been gradually used for genetic dissection of quantitative traits in fungi since the 2010s. However, such studies were mainly limited to the model fungi and fungal pathogens, such as *Saccharomyces cerevisiae* (Mehmood et al., 2011; Connelly and Akey, 2012), *Neurospora crassa* (Palma-Guerrero et al., 2013), *Fusarium graminearum* (Talas et al., 2012), and *Heterobasidion annosum* (Dalman et al., 2013).

In *L. edodes*, the genetic dissection of important agronomic traits through linkage mapping and association mapping has recently been performed in our laboratory (Xiang, 2015; Gong et al., 2016). Utilizing linkage mapping, Gong et al. (2016) mapped QTLs underlying seven morphological traits related to characteristics of fruiting body in *L. edodes*. Six QTL hotspot regions on six linkage groups (LGs) were detected across two segregating testcross populations. A preliminary study of association mapping in a *L. edodes* population containing 95 wild and one cultivated strains was conducted by using 95 Insertion-Deletion (InDel) and two simple sequence repeat (SSR) markers (Xiang, 2015). Twenty markers were detected to be significantly associated with 10 traits by the general linear model, with phenotypic variations explained by each marker (R^2^) ranging from 1.29% to 37.60%. However, in these pioneer studies, the phenotypes were only determined in a single environment, and the reliability of the marker-trait association remains to be confirmed.

In this study, 89 *L. edodes* cultivars collected from major producing areas in China were used to perform association mapping for 11 important agronomic traits by using 297 genome-wide InDel and SSR markers. Cultivation tests were conducted in 2013 and 2014. The objectives of this study were to (1) evaluate the phenotypic and genotypic diversities of *L. edodes* cultivars in China, (2) investigate QTLs associated with important agronomic traits, and (3) mine candidate genes associated with these traits. The vast number of polymorphic molecular markers identified here could provide a valuable resource for future studies on genetics and breeding in *L. edodes*. Findings of this study could also benefit MAS and help illustrate the genetic architecture of important agronomic traits in *L. edodes*.

## Materials and methods

### *Lentinula edodes* strains

A total of 89 *L. edodes* cultivars currently cultivated in main producing areas of China were used in this study (Supplementary Table S1). All the tested strains were either provided by research institutes or collected from different mushroom growing farms, and were preserved in the Institute of Applied Mycology, Huazhong Agricultural University (Wuhan, China, 114.35°E, 30.48°N).

### Phenotypic evaluation

Cultivation tests of the 89 *L. edodes* cultivars were performed in the Huazhong Agricultural University. All strains were allocated in a mushroom house in accordance with the randomized-block design with two blocks, and seven bags of each strain were contained in each replication. Phenotypes were measured from 10 fruiting bodies of each strain. Data were collected from August 2013 to May 2014, and August 2014 to May 2015. Phenotype evaluation of nine fruiting body-related traits was carried out as previously: PD, pileus diameter (mm); PT, pileus thickness (mm); PW, pileus weight (g); SL, stipe length (mm); SD, stipe diameter (mm); SW, stipe weight (g); NF, number of fruiting bodies (per/bag); WF, weight of a single fruiting body (g); Y, total weight of fruiting bodies per bag (g/bag) (Gong et al., 2014b). Two traits about MGRs on both the MYG medium (2% malt extract; 2% glucose; 2% agar; 0.1% peptone; 0.1% yeast extract) and the mixed sawdust (SD) medium (DGR-myg and DGR-sd) were also measured as previously (Gong et al., 2013, 2014a). A brief description of all the 11 traits is summarized in Table 1.

**Table 1:** **Statistical characteristics of the 11 agronomic traits of ***Lentinula edodes*****.

**Trait (unit)**	**Description^a^**	**Year**	**Minimum**	**Maximum**	**Mean**	**Range**	**Sd^b^**	**CV^c^(%)**	**r^d^**	***H^2^(%)***
DGR-myg (mm/d)	Mycelium growth rate in MYG medium	2013	1.47	6.32	5.51	4.85	0.77	13.99	/	98.82
DGR-sd (mm/d)	Mycelium growth rate in sawdust medium	2013	3.53	4.40	3.96	0.87	0.20	5.08	/	87.84
PD (mm)	Average diameter of pileus, determined as the mean of two perpendicular diameters	2013	29.54	65.48	45.67	35.94	8.92	19.54	0.398^**^	85.50
		2014	30.81	67.82	53.03	37.02	7.94	14.96		
PT (mm)	Average thickness of pileus	2013	5.92	15.39	9.81	9.47	2.19	22.30	0.491^**^	86.91
		2014	5.05	13.90	10.56	8.85	2.02	19.10		
PW (g)	Average weight of pileus	2013	2.80	30.36	11.77	27.56	6.82	57.96	0.420^**^	84.64
		2014	3.88	28.90	14.58	25.01	5.14	35.23		
SD (mm)	Average diameter of stipe	2013	5.95	17.46	10.03	11.52	2.55	25.43	0.513^**^	86.96
		2014	7.07	15.66	10.70	8.60	1.82	16.99		
SL (mm)	Average length of stipe	2013	23.09	63.67	38.45	40.58	8.12	21.11	0.544^**^	89.89
		2014	22.09	62.33	41.28	40.25	7.88	19.09		
SW (g)	Average weight of stipe	2013	0.75	15.34	3.94	14.59	2.89	73.34	0.487^**^	86.05
		2014	0.93	9.37	4.25	8.45	1.73	40.64		
NF (per/bag)	Average number of total fruiting bodies per bag during the whole harvest time	2013	2.29	88.25	22.13	85.96	17.39	78.57	0.555^**^	91.78
		2014	1.90	62.14	13.86	60.24	12.33	88.95		
WF (g/per)	Ratio of yield to the number of fruiting bodies harvested during the whole harvest time	2013	2.08	39.07	13.89	36.99	8.35	60.08	0.317^*^	84.15
		2014	4.10	35.95	17.63	31.84	6.58	37.30		
Y (g/bag)	Total weight of fruiting bodies per bag during the whole harvest time	2013	66.52	354.52	193.23	288.00	48.61	25.16	0.281^*^	81.30
		2014	45.62	310.57	172.35	264.95	61.43	35.64		

a *Descriptions of the 11 traits are cited from our previous reports with minor modification (Gong et al., 2013, 2014a,b)*.

b *Sd: standard deviation*.

c *CV: coefficient of variation, was calculated by dividing the standard deviation by the mean of each trait*.

d *Correlation coefficients (r) of the 11 traits between year 2013 and 2014*.

* *P < 0.05*;

** *P < 0.01*.

Descriptive statistics, analysis of variance (ANOVA) and correlation analysis were performed using SPSS 17.0 (SPSS Inc., Chicago, IL, USA). For each trait, the phenotypic data were subjected to normality tests (Kolmogorov-Smirnov test). The coefficient of variation (CV) of each trait was calculated by dividing the standard deviation by the mean. ANOVA was first performed with data for each trait in each year independently for the genotypic variation according to the model: *Y* = μ + *G* + ε, where μ is the mean value, *G* is the genotypic effect and ε represents the residual effect. Data from the two independent year experiments were then combined for analyses according to the model: *Y* = μ + *G* + *E* + *G* × *E* + ε, where μ is the mean value, *G* represents the genotypic effect, *E* is the year effect, *G* × *E* is the genotype and year interaction, and ε is the residual effect. Broad-sense heritability (*H*^2^) was assessed with the formula *H*^2^ = $\sigma_{G}^{2}$/[$\sigma_{G}^{2}$ + ($\sigma_{e}^{2}$/*n*)] for the two traits relevant to MGR, and *H*^2^ = $\sigma_{G}^{2}$/[$\sigma_{G}^{2}$ + ($\sigma_{G\times E}^{2}$/*nr*) + ($\sigma_{e}^{2}$/*n*)] for the nine fruiting body-related traits, where $\sigma_{G}^{2}$ is the genotypic variance, $\sigma_{e}^{2}$ is the error variance, $\sigma_{G\times E}^{2}$ is the variance of genotype and year interaction, *n* is the number of replicates in the experiment, and *r* is the number of years.

Phenotypic data in both years were averaged for each fruiting body-related traits. The means were then combined with the phenotypic data of MGRs to construct an Unweighted Pair Group Method with Arithmetic Averaging (UPGMA) dendrogram using the NTSYS-pc (version 2.10e) program (Rohlf, 2000).

### SSR and InDel genotyping

A total of 389 InDel and 104 SSR markers were developed according to our previous description based on the L54A reference genome of *L. edodes* (GenBank accession number: LOHM00000000) (Gong et al., 2016). Among these markers, two SSR and 166 InDel markers were employed in our previous studies (Xiang, 2015; Gong et al., 2016). PCR and electrophoresis in the SSR and InDel analyses were conducted as previously, with minor modifications (Xiang et al., 2016). Eight strains (Cr04, L135, S602, 430, 9508, Hunong-3, Qin02, and Shandong-1) were randomly selected to screen for the 389 InDel and 104 SSR markers. A total of 379 polymorphic markers, comprising 328 InDels and 51 SSRs, were then selected. Only 297 markers (249 InDels and 48 SSRs) with a minor allele frequency (MAF) of ≥0.05 and missing data ≤5% in the 89 strains were utilized for further analysis. These 297 markers were distributed in 241 scaffolds of the L54A reference genome (Supplemental Table S2).

### Genetic diversity analysis

Several genetic parameters were assessed to evaluate the genetic diversity of the tested strains. The observed number of alleles (*Na*), effective number of alleles (*Ne*), percentage of polymorphic loci (PPL), observed heterozygosity (*Ho*), expected heterozygosity (*He*), and Shannon's information index (*I*) were calculated by using POPGENE 1.32 (Yeh, 1997). Gene diversity (*H*) and polymorphism information content (PIC) were calculated via PowerMarker V3.25 (Liu and Muse, 2005).

### Analysis of population structure and differentiation

Three methods were used to infer the population structure of the 89 *L. edodes* cultivars. First, an unrooted neighbor-joining (NJ) tree was constructed using Powermarker V3.25. Second, Principal coordinate analysis (PCA) was conducted with GenAlEx version 6.501 (Peakall and Smouse, 2012). All 297 markers were utilized to compute the NJ tree and PCA. Third, STRUCTURE version 2.3 was employed to perform a Bayesian clustering analysis among our tested strains (Pritchard et al., 2010). To reduce the effect of LD between markers on the STRUCTURE results, only 241 markers that were distributed in different scaffolds were utilized in our analysis. The number of subgroups (*K*) was set from 1 to 15 to survey the optimal range of *K* using admixture model assumptions with correlated alleles. For each *K*, seven independent runs were performed, with a burn-in of 10,000 Markov Chain Monte Carlo (MCMC) iterations and a run length of 100,000. Then the Δ*K* method was used to identify the optimal value of *K* (Evanno et al., 2005). Strains assigned to the corresponding genetic groups were graphically visualized by Excel 2013. The genetic differentiation within and among populations and the *F*_*ST*_ values were measured by analysis of molecular variance (AMOVA) using GenAlex version 6.501 (Peakall and Smouse, 2012).

### Marker-trait association analysis

Linkage disequilibrium (LD) between loci and association mapping was analyzed using TASSEL 3.0 (Bradbury et al., 2007). The association between molecular markers and the 11 traits was analyzed with the mixed linear model (MLM). To reduce error from the population structure and kinship relationships, *Q*-matrix and *K*-matrix were generated by STRUCTURE version 2.3 and TASSEL 3.0, respectively. To control the false positive rate in association mapping, *P*-values were corrected with FDR (false discovery rate) using the method described by Benjamini and Hochberg (1995), which is widely used in association mapping (Cook et al., 2012; Slovak et al., 2014; Wen et al., 2014; Celik et al., 2016). A FDR-adjusted *P*-value cut-off of ≤0.05 was used to detect significant marker-trait associations. Annotated genes of the L54A reference genome within a ± 2 kb scope of the significant trait-associated markers were proposed as putative candidate genes for corresponding traits. Functions of these genes were annotated with the Blast2GO package (Conesa et al., 2005).

## Results

### Phenotypic variation

Because the 89 shiitake cultivars belong to different temperature types, eight strains were unable to fruit or produce an adequate number of fruiting body (>10) in 2 years' cultivation. Therefore, these eight strains were excluded from further analyses about fruiting body-related traits. Furthermore, 17 strains produced <10 fruiting bodies in 2013 or 2014. As a result, only 64 strains that produced >10 fruiting bodies both in 2013 and 2014 were used for two-way analysis of variance.

**Figure 1:**
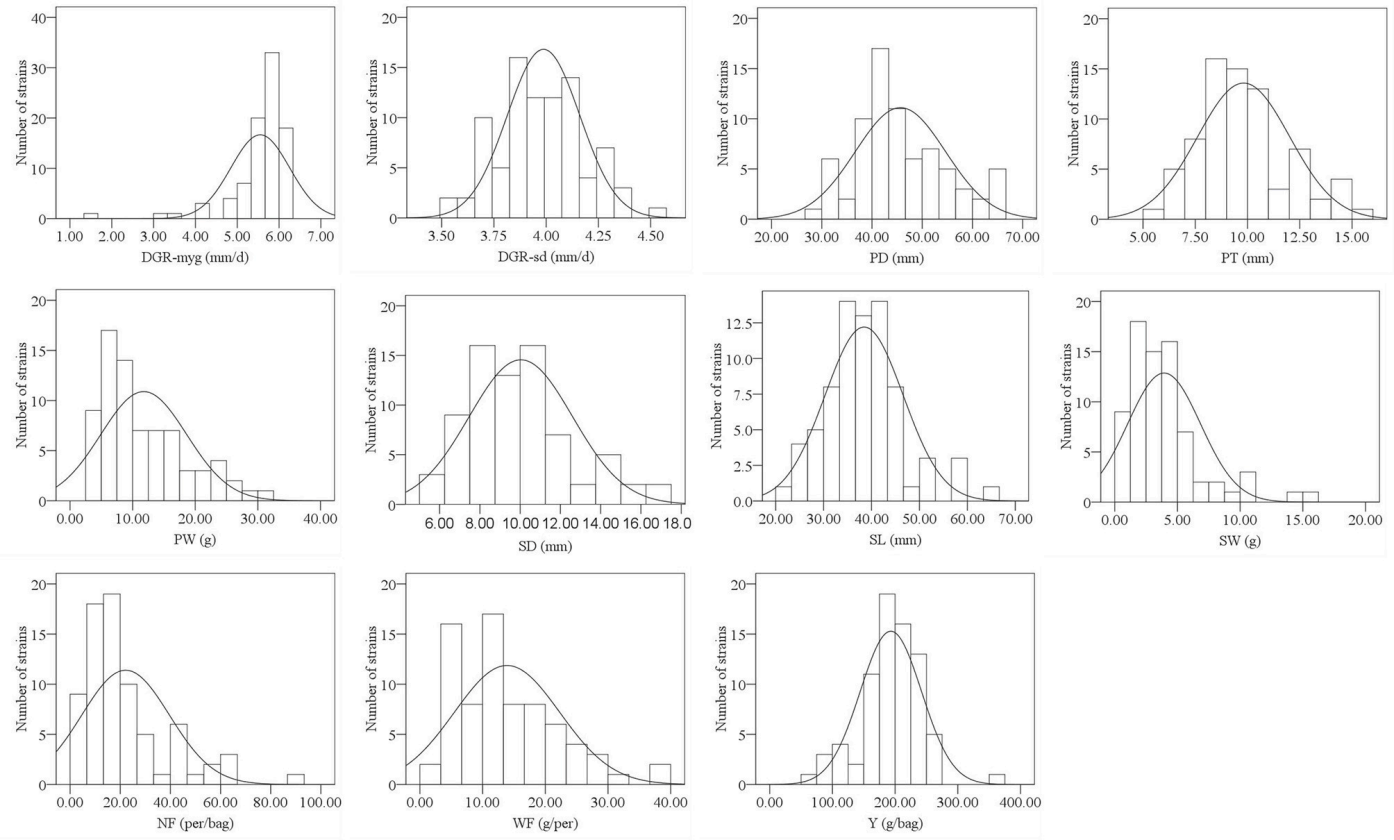
**Histograms showing the frequency distribution of 11 agronomic traits in 2013**. The y-axis denotes the number of strains, whereas the x-axis indicates value range of traits.

Frequency distribution of all surveyed agronomic traits was in the form of continuous variation (Figure 1, Supplementary Figure S1), suggesting that the traits were under quantitative and polygenic control. Most traits, except NF and DGR-myg, were distributed normally. After data transformation, the phenotypic data of NF and DGR-myg were still not fit the normal distribution. Thus, we used the original data of NF and DGR-myg for the statistical analysis.

For each trait, phenotypic variation among strains was confirmed by its range, mean, standard deviation, and CV (Table 1). The CV among strains ranged from 5.08% (DGR-sd) to 88.95% (NF) in 2014. For the nine fruiting body-related traits, the CV among strains ranged from 19.54% (PD) to 78.57% (NF) in 2013, and from 14.96% (PD) to 88.95% (NF) in 2014. In general, the CV of fruiting body-related traits were higher than those in mycelium-related traits. For the nine fruiting body-related traits, significant correlations (*P* < 0.05) were detected between experiments conducted in the 2 years (Table 1). As revealed by ANOVA analysis, genotype had highly significant effect on DGR-myg and DGR-sd (data not shown). Also, the effects of genotype, year, and interaction of genotype × year on the nine fruiting body-related traits were all significant (*P* < 0.05) (Supplementary Table S3). Values of the broad-sense heritability (*H*^2^) of the 11 agronomic traits were high, and varied from 81.30% (Y) to 98.82% (DGR-myg) (Table 1).

Pearson's correlation was used to uncover the relationship of the nine fruiting body-related traits in both years. There were significant positive correlations (*P* < 0.01) between six traits related to characteristics of single fruiting body, i.e., PD, PT, PW, SD, SL, and SW. Yield (Y) was significantly positively correlated with the yield-component trait NF but significantly negatively correlated with another yield-component trait WF (*P* < 0.01); NF was significantly negatively correlated with WF (*P* < 0.01; Table 2).

**Table 2:** **A matrix of Pearson correlation coefficients (***r***) for the 11 agronomic traits in Chinese ***Lentinula edodes*** cultivars**.

	**PD**	**PT**	**PW**	**SD**	**SL**	**SW**	**NF**	**WF**	**Y**
DGR-myg	0.125	0.211	0.223	0.223	0.142	0.197	−0.11	0.209	−0.208
DGR-sd	−0.054	0.004	−0.081	−0.014	−0.108	−0.133	0.07	−0.034	0.091
PD		0.853^**^	0.927^**^	0.852^**^	0.690^**^	0.756^**^	−0.733^**^	0.842^**^	−0.389^**^
PT	0.827^**^		0.813^**^	0.677^**^	0.525^**^	0.596^**^	−0.615^**^	0.748^**^	−0.403^**^
PW	0.884^**^	0.755^**^		0.883^**^	0.688^**^	0.828^**^	−0.711^**^	0.921^**^	−0.440^**^
SD	0.737^**^	0.753^**^	0.808^**^		0.780^**^	0.915^**^	−0.683^**^	0.864^**^	−0.275^*^
SL	0.761^**^	0.669^**^	0.654^**^	0.508^**^		0.851^**^	−0.629^**^	0.694^**^	−0.133
SW	0.821^**^	0.724^**^	0.864^**^	0.827^**^	0.840^**^		−0.601^**^	0.821^**^	−0.282^*^
NF	−0.850^**^	−0.835^**^	−0.776^**^	−0.737^**^	−0.734^**^	−0.750^**^		−0.767^**^	0.472^**^
WF	0.853^**^	0.746^**^	0.880^**^	0.710^**^	0.713^**^	0.834^**^	−0.780^**^		−0.475^**^
Y	−0.618^**^	−0.634^**^	−0.579^**^	−0.551^**^	−0.500^**^	−0.561^**^	0.731^**^	−0.561^**^	

*The abbreviations are the same as those in Table 1*.

* *P < 0.05*;

** *P < 0.01*.

*Pearson correlation coefficients are presented here in pairs for agronomic traits measured in 2013 (upper right triangle) and in 2014 (lower left triangle)*.

Sixty-four strains that we can get at least 10 fruiting bodies for each year were employed in phenotype-based UPGMA clustering. These strains were divided into two groups at the Euclidean distance of 118.19 (Supplementary Figure S2). Strains belonging to different groups in the NJ tree clustered together in the UPGMA dendrogram.

### Genetic diversity

A total of 873 alleles were detected from the 297 loci in all 89 strains, and the number of alleles in each locus varied from two to seven with a mean of 2.939 (Supplementary Table S2). The PIC value varied from 0.022 (S676_SSR2) to 0.731 (S95_ID5) with an average of 0.381. In all 89 strains, the average number of alleles from the 48 SSR markers was 3.229, higher than that from the 249 InDel markers (2.884). However, the PIC value of SSRs (0.379) was comparable to that of InDels (0.381), suggesting a similar genetic variation of both types of markers in the Chinese shiitake cultivars. From all 89 strains, the mean values of *Ne, He, I*, PPL, *H*, and PIC were 1.955, 0.456, 0.734, 100%, 0.454, and 0.381, respectively, indicating relatively low genetic variations among Chinese *L. edodes* cultivars. As for the two groups defined by the NJ tree, all the genetic parameters showed that the genetic diversity in Group A was lower than that in Group B (Table 3). The lower genetic diversity in Group A could be due to the smaller sample size.

**Table 3:** **Genetic variability for ***Lentinula edodes*** cultivars in China**.

**Population**	***Na***	***Ne***	***I***	***Ho***	***He***	***H***	**PIC**	**PPL%**
Total	2.939	1.955	0.734	0.545	0.456	0.454	0.381	100.00
Group A	2.162	1.622	0.500	0.444	0.327	0.319	0.262	85.19
Group B	2.582	1.847	0.672	0.580	0.428	0.424	0.352	99.66

*Observed number of alleles (Na), effective number of alleles (Ne), percentage of polymorphic loci (PPL), observed heterozygosity (Ho), expected heterozygosity (He), Shannon's information index (I), gene diversity (H), and polymorphism information content (PIC)*.

### Population structure and linkage disequilibrium

**Figure 2:**
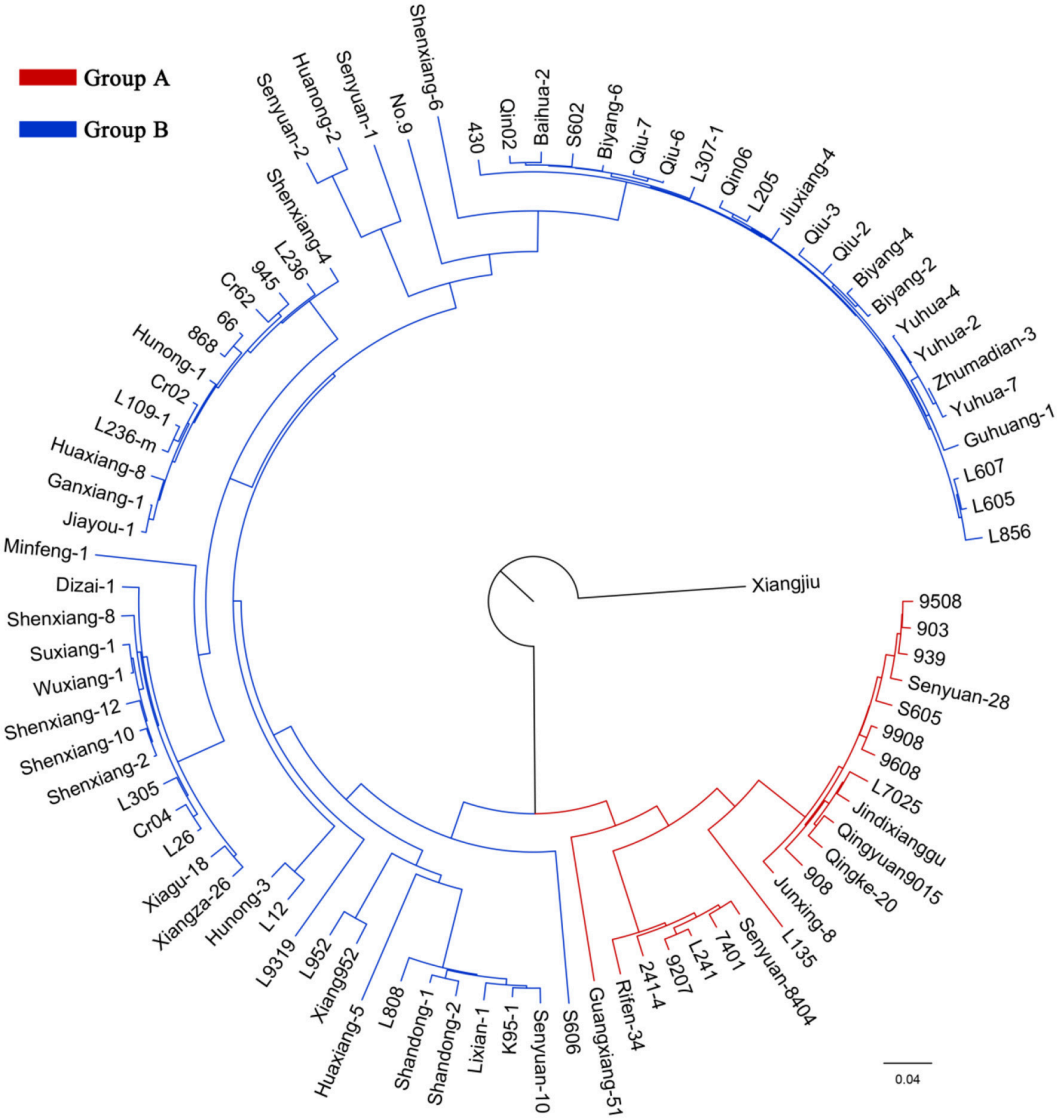
**A neighbor-joining tree of 89 ***Lentinula edodes*** cultivars**. The ancestors of the strains in the inferred groups are represented by different colors.

According to the genotyping data, the 89 strains represent 89 unique genotypes, and therefore are not clones. In the NJ tree of *L. edodes*, all strains except Xiangjiu clustered into two distinct groups (Figure 2). Group A consisted of 21 strains and Group B contained 67 strains. PCA also identified two groups congruent with those in the NJ tree (Supplementary Figure S3). The percentages of variation explained by the first 3 axes were 32.7%, 14.1%, and 9.8%.

Analysis of molecular variance (AMOVA) results suggested that the majority of genetic variation was included within populations (75.33%) (Table 4). The overall *F*_*ST*_ value across all the strains except Xiangjiu was 0.247, suggesting a great differentiation among *L. edodes* cultivars in China.

**Table 4:** **Analysis of molecular variance (AMOVA) among and within populations of ***Lentinula edodes*** cultivars in China**.

**Source**	**df**	**SS**	**MS**	**Est. Var**.	**%var**
Among populations	1	1337.155	1337.155	19.955	24.67
Within populations	174	10600.862	60.924	60.924	75.33
Total	175	11938.017		80.880	100.00

*df, degree of freedom; SS, sum of squared observations; MS, mean of squared observations; Est. var., estimated variance; %Var., percentage of total variance*.

**Figure 3:**
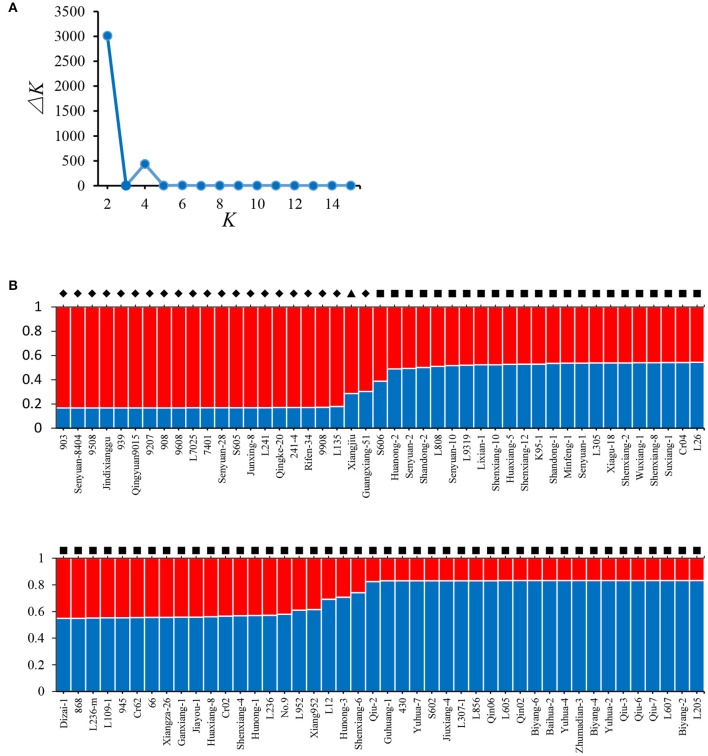
**Results of STRUCTURE analysis. (A)** Estimation of the number of populations for *K* ranging from 2 to 15 by Δ*K* values; **(B)** Classification of 89 *L. edodes* cultivars into two genetic groups. The distribution of the strains assigned to different groups is indicated by the color code (Group A: red, Group B: blue). The *y*-axis quantifies the cluster membership, and the *x*-axis lists the different strains. Strains from the different groups defined in the NJ tree are marked in different symbols: ♦, Group A; ■, Group B; ▴, Xiangjiu, excluded from the two groups in the NJ tree.

Model-based STRUCTURE was also utilized to investigate the population structure of the 89 strains. In the analysis of Δ*K*, a clear maximum was detected for *K* = 2 (Δ*K* = 3015) (Figure 3A). Therefore, two groups were identified in the collection of 89 *L. edodes* cultivars in China (Figure 3B), which agreed with the results of NJ tree and PCA. None of the 89 strains was assigned exclusively to one group or the other, and all strains shared mixed ancestries from the two groups. These demonstrate that the Chinese shiitake cultivars were genetically closely related.

A total of 19,122 (43.50%) InDel and SSR marker pairs displayed significant LD among all 89 strains (*P* ≤ 0.001). The *r*^2^ values among these marker pairs varied from 0.128 to 1, with an average of 0.316 (Supplementary Table S4). At the highly significant threshold of *r*^2^ ≥ 0.2, 30.43% (13,378) of the marker pairs remained in LD. In this study, owing to the fact that only 73 of these 297 makers were used to construct linkage map in our previous study (Gong et al., 2016), the genome-wide LD decay along with the increase of genetic distance were not detected (Supplementary Table S5). The averaged *r*^2^ was 0.419 in the region from 0 to 20 cM, and decreased to 0.384 in the region from 20 to 40 cM, then dropped to 0.361 in the region from 40 to 60 cM. However, the averaged *r*^2^ increased to 0.381 in the region of >60 cM. Extremely high LD levels were observed across all 89 strains. The average *r*^2^ of the unlinked marker pairs was 0.327, which was much lower than that in the linked marker pairs (0.400) (Supplementary Table S5).

### Association between traits and molecular markers

The numbers of strains used for association mapping are 75 (in 2013) and 69 (in 2014). Marker-trait associations for the 11 traits were evaluated using the *Q* + *K* model in Tassel 3.0 (Table 5). A total of 78 associations covering four traits (PD, PW, SW, and WF) were detected at FDR-adjusted *P* ≤ 0.05, and the phenotypic variation explained by each marker ranged from 12.07 to 31.32% (Table 5). In 2013 and 2014, the number of significant marker-trait associations were 17 and 61, respectively (Table 5). Eleven markers were associated with two traits (PD and PW) in 2013, and 41 markers were associated with four traits (PD, PW, SW, and WF) in 2014. The number of markers identified to be associated with the four traits varied from 9 (PD) to 26 (PW) with an average of 17.75.

**Table 5:** **Associations between agronomic traits and markers in the Chinese ***Lentinula edodes*** cultivars**.

**Year**	**Trait**	**Marker**	Scaffolds^*^	***P*^#^**	***P*_FDR**	***R*^2^ (%)**
2013	PD	S278_ID41^a^	Le_N7_S278	8.58 × 10^−5^	0.021	22.90
2013	PD	S328_ID5	Le_N7_S328	1.39 × 10^−4^	0.021	21.87
2013	PD	S278_ID10^a^	Le_N7_S278	2.95 × 10^−4^	0.024	20.51
2013	PD	S278_ID36	Le_N7_S278	4.4 × 10^−4^	0.024	19.32
2013	PD	S106_inID1	Le_N7_S106	4.7 × 10^−4^	0.024	15.53
2013	PD	S473_ID1	Le_N7_S473	5.07 × 10^−4^	0.024	19.25
2013	PD	S704_inID1^a^	Le_N7_S704	5.54 × 10^−4^	0.024	19.30
2013	PW	S278_ID41	Le_N7_S278	8.69 × 10^−5^	0.026	22.96
2013	PD	S127_ID1	Le_N7_S121	9.59 × 10^−4^	0.036	27.57
2013	PW	S328_ID5^a^	Le_N7_S328	2.4 × 10^−4^	0.036	20.79
2013	PW	S473_ID1	Le_N7_S473	3.6 × 10^−4^	0.036	20.09
2013	PW	S278_ID10^a^	Le_N7_S278	8.38 × 10^−4^	0.044	18.14
2013	PW	S127_ID1^a^	Le_N7_S121	8.76 × 10^−4^	0.044	28.02
2013	PW	S108_ID1	Le_N7_S108	9.52 × 10^−4^	0.044	20.70
2013	PW	S443_inID1	Le_N7_S443	0.001	0.044	13.69
2013	PW	S704_inID1^a^	Le_N7_S704	0.001	0.044	17.37
2013	PW	S613_inID1	Le_N7_S613	0.001	0.044	22.32
2014	WF	S278_ID10	Le_N7_S278	7.30 × 10^−6^	0.001	23.75
2014	WF	S427_ID1	Le_N7_S427	1.16 × 10^−5^	0.001	31.32
2014	WF	S560_ID1^b^	Le_N7_S560	1.26 × 10^−5^	0.001	25.96
2014	WF	S443_inID1	Le_N7_S443	1.45 × 10^−5^	0.001	22.05
2014	WF	S286_SSR3	Le_N7_S286	1.53 × 10^−5^	0.001	21.85
2014	WF	S278_ID36	Le_N7_S278	1.82 × 10^−5^	0.001	25.10
2014	WF	S458_ID5	Le_N7_S458	2.38 × 10^−5^	0.001	29.95
2014	WF	S488_ID1	Le_N7_S488	2.66 × 10^−5^	0.001	27.22
2014	WF	S551_inID1	Le_N7_S551	2.96 × 10^−5^	0.001	24.45
2014	WF	S603_ID1	Le_N7_S603	3.30 × 10^−5^	0.001	24.40
2014	WF	S267_ID1	Le_N7_S267	3.30 × 10^−5^	0.001	24.40
2014	WF	S106_inID1	Le_N7_S106	4.95 × 10^−5^	0.001	20.22
2014	WF	S636_inID1	Le_N7_S636	8.10 × 10^−5^	0.002	23.38
2014	WF	S255_ID1^c^	Le_N7_S255	1.04 × 10^−4^	0.002	24.47
2014	WF	S48_ID1^b^	Le_N7_S48	1.29 × 10^−4^	0.003	26.54
2014	WF	S131_ID1	Le_N7_S131	1.48 × 10^−4^	0.003	26.25
2014	PW	S560_ID1^b^	Le_N7_S560	1.67 × 10^−5^	0.005	27.95
2014	PW	S704_inID1^a^	Le_N7_S704	4.83 × 10^−5^	0.005	25.88
2014	PW	S211_ID1	Le_N7_S211	6.05 × 10^−5^	0.005	28.03
2014	PW	S488_ID1	Le_N7_S488	8.14 × 10^−5^	0.005	27.37
2014	PW	S767_ID1	Le_N7_S767	8.51 × 10^−5^	0.005	20.50
2014	PW	S255_ID1^c^	Le_N7_S255	1.33 × 10^−4^	0.007	26.29
2014	WF	S127_ID1	Le_N7_S121	4.03 × 10^−4^	0.007	26.36
2014	WF	S95_ID5	Le_N7_S95	5.11 × 10^−4^	0.008	28.33
2014	PW	S328_ID5^a^	Le_N7_S328	2.06 × 10^−4^	0.009	22.28
2014	WF	S641_SSR1	Le_N7_S641	6.94 × 10^−4^	0.011	27.25
2014	PW	S258_ID5_2	Le_N7_S258	3.10 × 10^−4^	0.011	27.03
2014	PW	S126_ID1	Le_N7_S126	3.29 × 10^−4^	0.011	21.08
2014	PW	S163_E1^c^	Le_N7_S163	5.19 × 10^−4^	0.015	28.67
2014	PW	S534_ID1	Le_N7_S534	5.68 × 10^−4^	0.015	25.57
2014	WF	S206_ID1	Le_N7_S206	0.001	0.017	24.25
2014	PW	S278_ID36	Le_N7_S278	8.19 × 10^−4^	0.020	19.12
2014	PW	S457_ID1	Le_N7_S457	9.42 × 10^−4^	0.021	29.09
2014	PW	S95_ID5	Le_N7_S95	9.80 × 10^−4^	0.021	29.38
2014	SW	S127_ID1	Le_N7_S121	3.98 × 10^−4^	0.024	29.51
2014	SW	S258_ID5_2	Le_N7_S258	4.00 × 10^−4^	0.024	26.97
2014	SW	S90_E1	Le_N7_S90	4.03 × 10^−4^	0.024	17.11
2014	SW	S278_ID33	Le_N7_S278	4.03 × 10^−4^	0.024	17.11
2014	SW	S553_ID5	Le_N7_S553	4.03 × 10^−4^	0.024	17.11
2014	SW	S108_ID1	Le_N7_S108	4.97 × 10^−4^	0.024	23.66
2014	PD	S278_ID41^a^	Le_N7_S278	1.81 × 10^−4^	0.025	20.71
2014	PD	S560_ID1^b^	Le_N7_S560	1.96 × 10^−4^	0.025	20.64
2014	PD	S278_ID10^a^	Le_N7_S278	3.31 × 10^−4^	0.025	16.92
2014	PD	S704_inID1^a^	Le_N7_S704	3.43 × 10^−4^	0.025	20.10
2014	PW	S278_ID3	Le_N7_S278	0.001	0.027	23.89
2014	WF	S178_ID1	Le_N7_S178	0.002	0.030	13.11
2014	WF	S704_inID1	Le_N7_S704	0.002	0.030	16.55
2014	SW	S32_E1	Le_N7_S32	7.16 × 10^−4^	0.030	28.04
2014	SW	S636_inID1	Le_N7_S636	8.54 × 10^−4^	0.031	19.22
2014	SW	S178_ID1	Le_N7_S178	0.001	0.033	15.25
2014	SW	S346_ID1^b^	Le_N7_S346	0.001	0.033	14.73
2014	SW	S470_ID1	Le_N7_S470	0.001	0.037	14.22
2014	PW	S127_ID1^a^	Le_N7_S121	0.002	0.039	24.68
2014	WF	S470_ID1	Le_N7_S470	0.003	0.039	12.07
2014	PW	S90_E1	Le_N7_S90	0.003	0.039	12.71
2014	PW	S278_ID33	Le_N7_S278	0.003	0.039	12.71
2014	PW	S553_ID5	Le_N7_S553	0.003	0.039	12.71
2014	PW	S278_ID10^a^	Le_N7_S278	0.003	0.040	12.78
2014	SW	S160_ID1	Le_N7_S160	0.002	0.044	13.48
2014	SW	S278_ID10	Le_N7_S278	0.002	0.044	13.75
2014	PW	S35_inID1	Le_N7_S35	0.003	0.045	24.87

a *Marker detected in both years*;

b *markers detected by Gong et al. (2016)*;

c *markers detected by Xiang (2015)*;

*P_FDR: P value after FDR correction*;

*R^2^: Phenotypic variation explained by each marker*;

* *Scaffolds names are derived from the L54A reference genome*.

# *Original P values detected by TASSEL 3.0*.

The association of a single marker with multiple traits could be the result of pleiotropy. Twenty-one molecular markers were associated with two to four traits (Table 6), five of which (S127_ID1, S328_ID5, S278_ID10, S278_ID41, and S704_inID1) were identified to be associated with the same trait in both years. For instance, S278_ID10 was found to be associated with PD and PW in 2013, and associated with WF, PD, PW, SW in 2014. In particular, associations between S278_ID10 and PD and PW were detected in both years.

**Table 6:** **Markers associated with at least two traits**.

**Marker**	**Number of associated traits**	**Traits^a^**
S106_inID1	2	PD (2013); WF (2014)
S108_ID1	2	PW (2013); SW (2014)
S127_ID1	4	PD, PW (2013); WF, SW, PW (2014)
S178_ID1	2	WF, SW (2014)
S255_ID1	2	WF, PW (2014)
S258_ID5_2	2	PW, SW (2014)
S278_ID10	4	PD, PW (2013); WF, PD, PW, SW (2014)
S278_ID33	2	SW, PW (2014)
S278_ID36	3	PD (2013); WF, PW (2014)
S278_ID41	2	PD, PW (2013); PD (2014)
S328_ID5	2	PW, WF (2014)
S443_inID1	2	PW (2013); WF (2014)
S470_ID1	2	SW, WF (2014)
S473_ID1	2	PD, PW (2013)
S488_ID1	2	WF, PW (2014)
S553_ID5	2	SW, PW (2014)
S560_ID1	3	WF, PW, PD (2014)
S636_inID1	2	WF, SW (2014)
S704_inID1	3	PD, PW (2013); PW, WF, PD (2014)
S90_E1	2	SW, PW (2014)
S95_ID5	2	PW, WF (2014)

a *Number in brackets indicates the year when the trait-marker associations were detected*.

Ninety-seven annotated genes were detected within a ± 2 kb scope of the significant trait-associated markers in the *L. edodes* reference genome, 31 of which contained trait-associated markers. Results from Blast2GO indicated that the 97 genes were involved in a wide range of molecular functions (level 3), including organic cyclic compound binding, small molecule binding, ion binding, transferase activity, carbohydrate derivative binding, hydrolase activity, heterocyclic compound binding, oxidoreductase activity, and protein binding (Supplementary Figure S4).

## Discussion

### Genetic variation of the 11 agronomic traits

In this study, ANOVA revealed the significant influences of the genotypes for all the surveyed traits. Heritability was also high for all the 11 traits. These observations indicated that the majority of the investigated traits were highly inheritable, and genotypic effect was the main factor to generate phenotypic variations.

The CV of the two mycelium growth-related traits were smaller than those of the nine fruiting body-related traits. This is because the two traits were determined in incubators and therefore not affected by the changing environmental conditions under which the nine traits were measured. Indeed, ANOVA revealed significant differences of the fruiting body-related traits between the 2 years, suggesting a strong effect of environmental factors (i.e., year) on these traits.

Here, we observed extensive significant correlations between the nine fruiting body-related traits of *L. edodes* cultivars, in agreement with previous results detected in two segregating populations and one natural population (Gong et al., 2014b). Yield and yield-component traits of *L. edodes* were found to exhibit the triangular relationship as displayed in our recent report (Gong et al., 2014b) and in *A. bisporus* (Foulongne-Oriol et al., 2012a), i.e., yield was positively correlated with NF but negatively correlated with WF; and NF was negatively correlated with WF. For nine fruiting body-related traits and two mycelium growth-related traits, no obvious correlation was observed in 2013, which indicated that the growth of mycelium and development of fruiting bodies may be independently controlled.

A total of 21 molecular markers were identified to be associated with two to four traits by association mapping. For instance, S106_inID1 was associated with four traits (WF, PD, PW, and SW), and S560_ID1 associated with three traits (WF, PW, and PD), partly illustrating the genetic basis of phenotypic correlation between these traits. The two major reasons for trait correlations are pleiotropy and close linkage between QTLs controlling different traits (Mackay et al., 2009; Chen and Lübberstedt, 2010). Our recent work on QTL mapping in two segregating populations also suggested that the co-localization of QTLs underlining different traits may be the genetic basis for phenotypic correlation of fruiting body-related traits in *L. edodes* (Gong et al., 2016). Combining evidences from both association mapping and our recent results from QTL mapping, we postulate that the genetic basis of phenotypic correlation in *L. edodes* is the tight linkage of QTLs and pleiotropy.

Multigenic effects were also observed in this study (Table 5). For instance, nine markers, including S278_ID10, S278_ID36, S278_ID41, and S328_ID5, were associated with PD. Multigenic effects suggested that these traits in *L. edodes* were complex quantitative traits that were affected by polygenes.

### Genetic diversity

Understanding the genetic diversity and genetic basis underlying important agronomic traits could improve breeding schemes of *L. edodes*. In general, the processes of domestication and breeding have a strong impact on the genetic diversity of cultivated species (Font i Forcada et al., 2015). Here, the values of Shannon's information index (*I*) and polymorphism information content (PIC) revealed by InDel and SSR markers were 0.734 and 0.381, respectively. In a wild population containing 88 Chinese *L. edodes* strains, the *I* and PIC values were 0.836 and 0.395, respectively (Xiang et al., 2016). In another study, the PIC value was 0.53 in 89 *L. edodes* strains from East Asia (Kim et al., 2009). Genetic variation of Chinese *L. edodes* cultivars is low and was postulated to be derived from a limited number of elite strains (Chiu et al., 1996). Therefore, the wild strains should be introduced into the breeding schemes to diversify the genetic basis of shiitake cultivars in China.

### Population structure and linkage disequilibrium

Detailed knowledge on population structure is important to control spurious associations between phenotypes and genotypes in association mapping (Pritchard et al., 2000). Model-based analysis of population structure could provide necessary information in association mapping. Here, population structure analyses based on three methods demonstrated that the Chinese *L. edodes* cultivars could be divided into two unique groups, with Xiangjiu being the sole exception. This strain was proven to be distinct from other *L. edodes* cultivars in previous clustering analyses (Fu et al., 2010; Liu et al., 2015). Using strains different from the current study, the *L. edodes* cultivars in China was also separated into two main groups (Zhang et al., 2007; Fu et al., 2010; Liu et al., 2012). Therefore, it is reasonable to speculate that *L. edodes* cultivars in China contained two different gene pools, which possibly resulted from domestication and breeding. Great genetic differentiation existed between the two groups as indicated by a *F*_*ST*_ value of 0.247, which is comparable to that in the Chinese wild *L. edodes* population (*F*_*ST*_ = 0.252) (Xiang et al., 2016).

A high level of LD among marker pairs was observed in this study. As mentioned before, the narrow genetic base of Chinese shiitake cultivars revealed here and in previous studies (Chiu et al., 1996; Fu et al., 2010) might be one of the factors that could explain the high level of pairwise LD. Also, a small number of tested strains and molecular markers may cause bias of LD estimates. Furthermore, the population structure contributes to increasing LD level. Population structure could create unexpected LD between unlinked loci across the genome (Yan et al., 2011). The mixing of individuals belonging to different subpopulations with different allele frequencies creates LD, when these subpopulations are admixed to construct a panel of lines for association mapping. Significant LD between unlinked loci results in false-positive associations between a marker and a trait (Soto-Cerda and Cloutier, 2012). In this study, small structured population with narrow genetic base may be the major factors that causes the high level LD and then led to spurious associations between marker alleles and the phenotypes. Therefore, the further association studies require the careful choice of germplasm, as well as a larger number of markers and strains.

### Association mapping

Association mapping has been successfully utilized in crop species, such as maize, cotton, wheat, and rice (Abdurakhmonov and Abdukarimov, 2008). Due to their edible and medicinal values, mushrooms have been consumed by humans for a long time. However, linkage and association mapping in mushroom species are still in their infancy, and information is limited to identify QTLs controlling agronomic traits in mushroom species. Here, we detected 78 marker-trait associations covering 43 molecular markers and four traits. Marker-trait associations detected by this method could provide valuable information for MAS in breeding schemes of *L. edodes*.

Among the 297 markers used here, 73 markers were the same as those used by Gong et al. (2016), while 47 markers were employed by Xiang (2015). Five markers were found to be associated with the same traits as in previous reports (Xiang, 2015; Gong et al., 2016) (Table 5). Gong et al. (2016) revealed that marker S48_ID1 was located in a QTLs-hotspot region in LG2 that was related to PD, PT, PW, SL, SD, SW, and WF. In this study, S48_ID1 was also identified to be associated with WF. S560_ID1 was significantly associated with WF, PW and PD, consistent with previous findings (Gong et al., 2016). S346_ID1 was found to be associated with SW and lie in a QTL hotspot region in LG4 related to SW, SL, and SD (Gong et al., 2016). Three of the six hotspot regions previously identified by Gong et al. (2016) were confirmed by this association mapping, thus suggesting that there are reliable regions harboring QTLs related to fruiting body in *L. edodes*. Moreover, S255_ID1 was found to be associated with PW and WF, and S163_E1 associated with PW, in agreement with a previous report (Xiang, 2015).

Five markers were identified to be associated with the same trait in both years. They are S127_ID1 and S328_ID5 for PW, S278_ID10 and S704_inID1 for PW and PD, and S278_ID41 for PD. It is worth mentioning that S278_ID41 is located in a <1 kb position to S278-R/F that lay in a QTL hotspot in MG4 special for SL, PD, and PW (Gong et al., 2016). Hence, S278_ID41 is also located in this QTL hotspot in MG4.

The foregoing 10 markers were verified by previous studies or detected in both years, suggesting that association mapping in shiitake cultivars can effectively identify major QTLs underlying agronomic traits. Apart from the five markers that were previously detected, 38 markers related to four traits in *L. edodes* were newly reported here, demonstrating that association mapping could detect more trait-associated markers than conventional linkage mapping. Moreover, unlike linkage mapping, which is based on a bi-parental population, association mapping investigates genetic variations in a natural population, and thus can evaluate many alleles simultaneously (Abdurakhmonov and Abdukarimov, 2008). Therefore, this study confirmed the feasibility and reliability of association mapping in *L. edodes*.

The 10 aforementioned markers resided on or were close to 24 annotated genes of the *L. edodes* reference genome (Supplementary Table S6). These genes could be potential candidate genes related to agronomic traits. By checking the RNA-seq expression levels of the 24 candidate genes in shiitake strain L54 (unpublished data), 13 of them (54.17%) were found to be significantly up-regulated or down-regulated during the transition from mycelium to primordium, with fold changes >2 (Supplementary Figure S5). Hence, it is reasonable to speculate that most of these candidate genes are involved in the development of fruiting body.

Despite the importance of edible mushrooms, research on their breeding and production are still very limited as compared to other crops, which may be partly due to the lack of knowledge of their genetics and breeding system (Chakravarty, 2011). Linkage mapping is usually conducted in purpose-created segregating populations, such as progeny of selected parents. However, the resolution of linkage mapping is hampered by the limited number of recombination in the segregating population. Moreover, there are some particularities in linkage mapping in edible mushrooms. Construction of genetic map is performed using a haploid progeny, whereas the phenotypic evaluation of some traits is only possible at the dikaryotic stage, after crossing haploid progenies with the compatible monokaryotic tester (Foulongne-Oriol, 2012). For each locus, these mushrooms have the same allele from the tester line in one nucleus and contain the allele from one of the parents in the other nucleus (Gao et al., 2015). The identified QTLs reflect the allelic substitution effect of the segregating allele with their interactions with the constant allele from the tester nucleus, and such constraints may lead to inconsistencies in QTL detection (Foulongne-Oriol, 2012). Alternatively, association mapping compares favorably to linkage mapping for the dissection of natural variation by using diverse germplasms, such as those derived from the wild populations, germplasm collections or subsets of breeding germplasm (Zhu et al., 2008). Because many generations have passed and more recombinations occurred, the resolution of association mapping is considerably higher than that in simple bi-parental populations (Rafalski, 2010). However, the complex population structure is one of the sources of false positives in association mapping. For instance, the complex and heterogeneous population structure of a *S. cerevisiae* population was reported to lead to a high type I error rate in association mapping (Connelly and Akey, 2012). A discernable population structure was also reported in shiitake cultivars here. Moreover, the allele frequency distribution has considerable impact on the detection power of association mapping. Here, only the markers with a (MAF) ≥0.05 were utilized for association analysis.

Overall, the combination of linkage mapping and association mapping could be a more powerful strategy for dissecting the genetic architectures of quantitative traits in edible mushrooms. To begin with, the genomic regions underlying quantitative traits of interest could be defined by linkage mapping. Then, based on the results of linkage mapping, fine mapping could be performed by candidate gene-based association analysis to identify the loci or genes for the traits of interest. In addition, the utilization of nested association mapping populations (Yu et al., 2008) could be a promising approach for genetic dissection of quantitative traits in edible mushrooms.

## Conclusion

In summary, we reported here the genetic diversity, population structure and association mapping of agronomic traits in a Chinese *L. edodes* population containing 89 cultivars by using 297 genome-wide markers. A narrow genetic base with a discernable population structure was observed in the Chinese shiitake cultivars. In association mapping, a total of 43 markers were detected to be significantly associated with four traits. Five of these marker-trait associations were verified by previous studies and another five of them were significantly detected in cultivation tests performed in two consecutive years. Our results have highlighted the significant potential of LD-based association mapping of complex agronomic traits in shiitake with consideration of the population structure. Associations identified here could provide insights into the genetic architecture of important agronomic traits, thus paving a way toward implementation of MAS in *L. edodes*.

## Author contributions

YX and YB conceived and designed the experiments; CL and LZ performed the experiments; WN and HK developed the molecular markers; CL, WG, and ZY analyzed the phenotypic and genotypic data; YX, WG, and MC wrote the paper.

### Conflict of interest statement

The authors declare that the research was conducted in the absence of any commercial or financial relationships that could be construed as a potential conflict of interest.
