# Supplementary material for: Association Mapping Reveals Genetic Loci Associated with Important Agronomic Traits in Lentinula edodes, Shiitake Mushroom
Source: Front Microbiol. 2017 Feb 17;8:237. doi: 10.3389/fmicb.2017.00237 (PMC5314409; doi:10.3389/fmicb.2017.00237)
Supplement: Supplementary file 1 [file Table1.doc]

Supplementary Material

# Association mapping reveals genetic loci associated with important agronomic traits in *Lentinula edodes*, shiitake mushroom

Chuang Li†, Wenbing Gong†, Lin Zhang, Zhiquan Yang, Wenyan Nong, Yinbing Bian, Hoi-Shan Kwan, Man-Kit Cheung, Yang Xiao*

*** Correspondence:** Yang Xiao xiaoyang@mail.hzau.edu.cn; xyfungi@163.com

**Supplementary Table S1.** **Cultivated strains of *Lentinula edodes* used in this study.**

| Origin ^a^ | Number of strains | Strain names |
| --- | --- | --- |
| Biyang | 8 | Biyang-2, Biyang-4, Biyang-6, Guhuang-1, Yuhua-2, Yuhua-4, Yuhua-7, Zhumadian-3 |
| Chengdu | 1 | Jindixianggu |
| Fujian-1 | 1 | Dizai-1 |
| Fujian-2 | 2 | Jiayou-1, L236-m |
| Guangdong | 3 | Guangxiang-51, Xiangjiu, Xiangza-26 |
| Henan-1 | 2 | K95-1, Rifen-34 |
| Henan-2 | 3 | 908, 9608, 9908 |
| Hubei | 1 | 945 |
| Jiangsu | 1 | Suxiang-1 |
| Lingbao | 1 | Lixian-1 |
| Lishui | 2 | L808, L9319 |
| Nanchang | 1 | Ganxiang-1 |
| Qingyuan | 3 | 241-4, Qingke-20, Qingyuan9015 |
| Sanming-1 | 11 | 66, Cr02, Cr04, Cr62, L12, L135, L236, L241, L26, L856, Minfeng-1 |
| Shandong | 2 | Shandong-1, Shandong-2 |
| Shanghai | 11 | Hunong-1, Hunong-3, S602, S605, S606, Shenxiang-10, Shenxiang-12, Shenxiang-2, Shenxiang-4, Shenxiang-6, Shenxiang-8 |
| Suizhou-1 | 1 | No.9 |
| Suizhou-2 | 1 | Jiuxiang-4 |
| Suizhou-3 | 2 | Qin02, Qin06 |
| Suizhou-4 | 1 | 9508 |
| Wuhan | 21 | 430, 903, 7401, Baihua-2, Huanong-2, Huaxiang-5, Huaxiang-8, L109–1, L205, L305, L307–1, L605, L607, L7025, L952, Qiu-2, Qiu-3, Qiu-6, Qiu-7, Xiagu-18, Xiang952 |
| Wuyi | 1 | Wuxiang-1 |
| Yichang-1 | 5 | Senyuan-1, Senyuan-10, Senyuan-2, Senyuan-8, Senyuan-8404 |
| Yichang-2 | 1 | 9207 |
| Zhejiang-1 | 2 | 868, Junxing-8 |
| Zhejiang-2 | 1 | 939 |

^a^ Shanghai, Shanghai Academy of Agricultural Sciences; Qingyuan, Qingyuan Edible Fungi Scientific Research Center, Zhejiang Province; Suizhou-1, mushroom growing farm in Sanligang Town, Hubei Province; Suizhou-2, Suizhou Changjiu Mushroom Company, Hubei Province; Suizhou-3, mushroom growing farm in Caodian Town, Hubei Province; Suizhou-4, mushroom growing farm in Hongshan Town, Hubei Province; Sanming-1, Sanming Mycological Institute, Fujian Province; Sanming-2, Sanming Food Industry Institute, Fujian Province; Wuhan, Huazhong Agricultural University, Hubei Province; Lishui, Lishui Dashan Mushroom Research and Development Company, Zhejiang Province; Fujian-1, mushroom growing farm in Changting County, Fujian Province; Fujian-2, mushroom growing farm in Gutian County, Fujian Province; Nanchang, Jiangxi Academy of Agricultural Sciences, Jiangxi Province; Biyang, mushroom growing farm in Biyang County, Henan Province; Guangdong, Guangdong Institute of Microbiology, Guangdong Province; Chengdu, Sichuan Academy of Agricultural Sciences, Sichuan Province; Zhejiang-1, Zhejiang Academy of Forestry Sciences, Zhejiang Province; Zhejiang-2, mushroom growing farm in Qinyuan County, Zhejiang Province; Henan-1, Chengguan mushroom growing farm in Lushan County, Henan Province; Henan-2, mushroom growing farm in Xixia County, Henan Province; Yichang-1, Hubei Senyuan Mushroom Company, Hubei Province; Yichang-2, mushroom growing farm in Yuanan County, Hubei Province; Shandong, mushroom growing farm in Zibo City, Shandong Province; Jiangsu, Jiangsu Institute of Microbiology, Jiangsu Province; Wuyi, Wuyi Mycological Institute, Zhejiang Province; Hubei, mushroom growing farm in Huangpi District, Hubei Province.
