# Supplementary material for: Association Mapping Reveals Genetic Loci Associated with Important Agronomic Traits in Lentinula edodes, Shiitake Mushroom
Source: Front Microbiol. 2017 Feb 17;8:237. doi: 10.3389/fmicb.2017.00237 (PMC5314409; doi:10.3389/fmicb.2017.00237)
Supplement: Supplementary file 2 [file Table2.doc]

**Supplementary Table S2.** **Sequence information of polymorphic InDel and SSR primers used in this study**.

| Primer name | Scaffold | Start(bp) | End (bp) | Product size (bp) | Forward primer sequence (5' to 3') | Reverse primer sequence (5' to 3') | Marker Type | *H* | PIC | *Ne* |
| --- | --- | --- | --- | --- | --- | --- | --- | --- | --- | --- |
| S283_SSR1 | Le_N7_S283 | 48195 | 48210 | 168 | ATTTAGCAACAAAGCACGGG | CGAGTGCTGGACTGGATGTA | SSR | 0.612 | 0.532 | 2.576 |
| S357_SSR1 | Le_N7_S357 | 41460 | 41474 | 276 | AGGATCTGCGCCTCAGTAGA | AGAGGTCGTCGTCAAGCCTA | SSR | 0.467 | 0.358 | 1.875 |
| S490_SSR1 | Le_N7_S490 | 27941 | 27958 | 222 | CAAGTACGGCGTTCTCAACA | ACGATGGTAGCAGTCATCCC | SSR | 0.165 | 0.155 | 1.198 |
| S609_SSR1 | Le_N7_S609 | 13502 | 13519 | 230 | ACCAACACTCTTGTCCCGTC | ACGCTTAAGAGTAACGCCGA | SSR | 0.500 | 0.375 | 2.000 |
| S689_SSR1 | Le_N7_S689 | 108907 | 108921 | 273 | CTCTGGAGAAACCAGCCAAG | GTGTGTTTGCGGCCTTTAAT | SSR | 0.182 | 0.165 | 1.222 |
| S676_SSR2 | Le_N7_S676 | 123898 | 123915 | 187 | GGTTGCCAACTCATTTGGAT | ATGTCGCCATCGGTTTCTAC | SSR | 0.022 | 0.022 | 1.023 |
| S482_SSR1 | Le_N7_S482 | 67087 | 67098 | 282 | AATATCGCAGGAGGTTGTGG | GTACCGAAGCGAGCAAGTTC | SSR | 0.427 | 0.336 | 1.745 |
| S461_SSR1 | Le_N7_S461 | 11094 | 11119 | 226 | CCAAGAATTGAACGGGAAAA | ACATTAACGGCCCATTGCTA | SSR | 0.685 | 0.620 | 3.173 |
| S481_SSR1 | Le_N7_S481 | 34497 | 34514 | 285 | GACGTCTCTCTGGAGGATGC | CTCCTTTTCTCGTTCCCGTT | SSR | 0.486 | 0.436 | 1.945 |
| S631_SSR2 | Le_N7_S631 | 31670 | 31694 | 240 | CCCCATTAAGGAGGCATGTA | AGGGTCCCTTCCAACCTTAG | SSR | 0.383 | 0.310 | 1.622 |
| S60_SSR1 | Le_N7_S60 | 7416 | 7439 | 266 | TTATCGGAGACACAGGTCCC | TTCTTCTTCATTCGGGATGG | SSR | 0.260 | 0.226 | 1.351 |
| S158_SSR2 | Le_N7_S158 | 40116 | 40127 | 274 | TTCTTTGGGTGTGTCAACGA | CCTGAAATGGGAGGTTCTCA | SSR | 0.077 | 0.074 | 1.084 |
| S203_SSR1 | Le_N7_S203 | 184552 | 184569 | 242 | ACCATCACCACTTCCTCCTG | ATCCTCAAACCGCACAAAAG | SSR | 0.430 | 0.343 | 1.756 |
| S166_SSR2 | Le_N7_S166 | 110810 | 110827 | 270 | TTGCGAAAAGCAAGGAGAGT | TGTAGAGGGAGGCTGAGGAA | SSR | 0.033 | 0.033 | 1.034 |
| S380_SSR1 | Le_N7_S380 | 49712 | 49731 | 265 | CTTGCCACATTCCGATCTCT | CTTCTCCTTTCGCTGTTCCA | SSR | 0.504 | 0.454 | 2.015 |
| S411_SSR1 | Le_N7_S411 | 48309 | 48322 | 171 | CGTCGCTGTTTGGTGAATTA | CCCACAGATGTTTCCGAGTT | SSR | 0.154 | 0.142 | 1.183 |
| S399_SSR1 | Le_N7_S399 | 23887 | 23904 | 275 | TCTAATGGGGAAGTTGCAGG | CAAGTTTCTTGCGTTGACGA | SSR | 0.503 | 0.382 | 2.012 |
| S462_SSR1 | Le_N7_S462 | 179231 | 179248 | 253 | TGACTCCGTAAAATCCTGGC | CGTTCACAATGACCCAACTG | SSR | 0.694 | 0.637 | 3.270 |
| S508_SSR1 | Le_N7_S508 | 11252 | 11266 | 281 | GACGGCGGAGCTGTAGTATC | ACCAAACAACCTCCTCATCG | SSR | 0.555 | 0.466 | 2.248 |
| S6_SSR1 | Le_N7_S6 | 52458 | 52469 | 196 | GGGGCTACAAGCACATTCAT | GCGTAACTCGCACTGTGAAA | SSR | 0.670 | 0.613 | 3.031 |
| S218_SSR1 | Le_N7_S218 | 30777 | 30792 | 279 | CGGAGTCCAAGATAGGCAAG | GGCACAAGCGATAGGATGAT | SSR | 0.537 | 0.463 | 2.162 |
| S38_SSR2 | Le_N7_S38 | 53997 | 54011 | 215 | TGACAGTGTTTCCTTCTGCG | CGGGGACTTTCAACCAGTAA | SSR | 0.184 | 0.171 | 1.225 |
| S401_SSR1 | Le_N7_S401 | 28502 | 28522 | 291 | ATGGGGTAGAGCAACACGAC | CCGATCGACTCACCTCTGTT | SSR | 0.598 | 0.546 | 2.490 |
| S366_SSR1 | Le_N7_S366 | 77276 | 77339 | 294 | TACAATTGCCGCATCTTTTG | GTCCACTCGGAAAATCGGTA | SSR | 0.626 | 0.552 | 2.676 |
| S27_SSR1 | Le_N7_S27 | 53116 | 53129 | 265 | AATGTCGAGTGTTGGGCTTC | AAGACCGAAAGTGGAGCTGA | SSR | 0.646 | 0.582 | 2.827 |
| S175_SSR2 | Le_N7_S175 | 140250 | 140282 | 284 | TCACGATGTGCAGAAGAAGG | CGAACTTGGAGGAAGCAGTC | SSR | 0.500 | 0.375 | 1.999 |
| S267_SSR1 | Le_N7_S267 | 5342 | 5361 | 213 | GCCAGTGAGTGGGATCGTAT | ATCTTGAGTCGTCCACCCAG | SSR | 0.639 | 0.564 | 2.769 |
| S600_SSR1 | Le_N7_S600 | 29124 | 29138 | 254 | TGCAAGTTTTCCTTGAACCA | TTTGACTCTGCTCTTTGCCA | SSR | 0.494 | 0.372 | 1.975 |
| S579_SSR2 | Le_N7_S579 | 28775 | 28835 | 297 | CATCCTGACCTGCAAATCCT | ACACTCTCAAGCCTCTTCGC | SSR | 0.657 | 0.616 | 2.915 |
| S163_SSR1 | Le_N7_S163 | 60838 | 61049 | 212 | TATTGTCCTGGTGTGGCAAA | TAGAAAGACGCCGCTTTGAT | SSR | 0.504 | 0.383 | 2.017 |
| S23_SSR1 | Le_N7_S23 | 166762 | 166779 | 243 | TACCTCGTGCGGACTTTGAT | CATTGCTCGGATCCTTCATT | SSR | 0.484 | 0.367 | 1.937 |
| S220_SSR1 | Le_N7_S220 | 151367 | 151384 | 226 | GCAAGAGGCAGATTACGAGC | TTCACGTGAGTGAAGATGCC | SSR | 0.304 | 0.261 | 1.437 |
| S764_SSR1 | Le_N7_S764 | 10937 | 10957 | 166 | CCACGAGAAGGCGTAGTAGC | TTGATGGGTGGAGGGAATTA | SSR | 0.581 | 0.492 | 2.385 |
| S63_SSR3 | Le_N7_S63 | 140477 | 140497 | 275 | TACTGTGGAAAGCGGAAACC | ATACTTTGCGAGTCGAGCGT | SSR | 0.428 | 0.395 | 1.749 |
| S370_SSR3 | Le_N7_S370 | 95326 | 95337 | 211 | TATGGAAGGTAGCATTGCCC | TCTCCTCTGCCCTGAGATGT | SSR | 0.335 | 0.279 | 1.503 |
| S438_SSR3 | Le_N7_S438 | 106488 | 106503 | 262 | CCGAGCACATCTACGTCAAA | TTCTGCGCCTAATGGTTCTC | SSR | 0.568 | 0.506 | 2.314 |
| S525_SSR3 | Le_N7_S525 | 60878 | 60905 | 296 | TGTAACACAGCGTCATGGGT | GAGGGGAGATGAGACGCATA | SSR | 0.642 | 0.595 | 2.794 |
| S388_SSR1 | Le_N7_S388 | 172600 | 172614 | 164 | AGCTACTGGAAGGACAGGCA | AAGGATCATCTGTCCAACGG | SSR | 0.426 | 0.380 | 1.741 |
| S286_SSR3 | Le_N7_S286 | 20770 | 20787 | 269 | TTGACCAAGCCATGACCATA | CTGGAGCCAATGTCGTAGGT | SSR | 0.135 | 0.126 | 1.157 |
| S172_SSR3 | Le_N7_S172 | 29285 | 29304 | 175 | GACTCCAGCTGCGCTAAGAT | CGGCGTGTTAATTGGGTACT | SSR | 0.475 | 0.391 | 1.903 |
| S327_SSR3 | Le_N7_S327 | 17505 | 17518 | 293 | TGGCAACAACTCTTACGCTG | CGTATTGGCCTGTCAGAGGT | SSR | 0.447 | 0.347 | 1.808 |
| S107_SSR1 | Le_N7_S107 | 41961 | 41978 | 274 | AAACGAAAACAGAGAGGCGA | GTGGTGACATGTACGTTCGG | SSR | 0.614 | 0.536 | 2.591 |
| S163_SSR3 | Le_N7_S163 | 61008 | 61021 | 212 | TATTGTCCTGGTGTGGCAAA | TAGAAAGACGCCGCTTTGAT | SSR | 0.528 | 0.417 | 2.120 |
| S170_SSR1 | Le_N7_S170 | 18949 | 18963 | 219 | TTTCATCTTCTGTCGTTGCG | ATCGGCTTTTGGTCTTGATG | SSR | 0.240 | 0.211 | 1.316 |
| S306_D1 | Le_N7_S306 | 16216 | 16398 | 183 | GAGAGGGCGATGAATGAGAG | ACTTCGAATCTTTCGTGCGT | InDel | 0.463 | 0.356 | 1.862 |
| S292_D1 | Le_N7_S292 | 13816 | 14041 | 226 | CTGTAGCACTTACGCACGGA | ATTGTCCTGGATTCGCACTC | InDel | 0.661 | 0.594 | 2.947 |
| S240_D1 | Le_N7_S240 | 157735 | 158003 | 269 | GCTGTTAGGGAGGGAAGAGC | ACTGCCTGCTTGGTTGAACT | InDel | 0.249 | 0.218 | 1.332 |
| S504_D1 | Le_N7_S504 | 5820 | 6104 | 285 | GCAATCGAGACTGATGCAAA | TCTCTAGGGACGCAGTCGAT | InDel | 0.493 | 0.382 | 1.972 |
| S462_D1 | Le_N7_S462 | 159458 | 159623 | 166 | GGTGAAACGAACCTGGAAAA | CAACGATCTTTGGTGGGAGT | InDel | 0.496 | 0.373 | 1.984 |
| S253_D2 | Le_N7_S253 | 75211 | 75400 | 190 | ACGACATCACCAATGCTTCA | TCAGGCGACACCTGTCTATG | InDel | 0.316 | 0.266 | 1.462 |
| S480_D1 | Le_N7_S480 | 7998 | 8232 | 235 | AGGAGAAGGAGATTGGGGAA | TGTTAGAGAAATGGGCAGGG | InDel | 0.439 | 0.343 | 1.784 |
| S243_D2 | Le_N7_S243 | 73293 | 73586 | 294 | TAACGGCTTCTGTCAGGCTT | GAATTCGCTCGAGTCCTTTG | InDel | 0.443 | 0.345 | 1.796 |
| S384_D1 | Le_N7_S384 | 35988 | 36255 | 268 | CACATGTTCCTGTGGATTCG | GGTGAAGGGACGAGTGGTAA | InDel | 0.430 | 0.343 | 1.756 |
| S395_D1 | Le_N7_S395 | 97883 | 98093 | 211 | GTGGAAGCCAATCTTCGTGT | TTGAGCTTTCCCAGCAGTCT | InDel | 0.488 | 0.374 | 1.953 |
| S143_E1 | Le_N7_S143 | 13427 | 13575 | 149 | GAAACTTGAAGCCGTTCGAC | GGATAAACAAGCGCGAGAAG | InDel | 0.495 | 0.373 | 1.982 |
| S313_E1 | Le_N7_S313 | 10852 | 11079 | 228 | CAACAGCGTTTTGTGGAATG | CCCCAAATTCCTCAGCTACA | InDel | 0.457 | 0.353 | 1.843 |
| S435_E1 | Le_N7_S435 | 59361 | 59537 | 177 | ACAGCCTGAACTGCTACCGT | CCCTTTCCACAGTGTCTGGT | InDel | 0.499 | 0.374 | 1.996 |
| S32_E1 | Le_N7_S32 | 5140 | 5388 | 249 | GTAATCATCGCGCCTTTTTC | AGCGAGCTATGACGACGATT | InDel | 0.482 | 0.402 | 1.929 |
| S384_E1 | Le_N7_S384 | 21792 | 21943 | 152 | AGCATTTCGTGCCCATATTC | GTCACCTTCGACGATATGCC | InDel | 0.568 | 0.503 | 2.316 |
| S442_E1 | Le_N7_S442 | 81826 | 82103 | 278 | CAATTGAGTGTTCGCACCAT | CCAGCAGTTGTCACTTCGTC | InDel | 0.500 | 0.386 | 2.002 |
| S217_E1 | Le_N7_S217 | 55246 | 55496 | 251 | AACCACGGTTCCTGAATGAC | TCTACACTTTCCCGACACCC | InDel | 0.334 | 0.285 | 1.501 |
| S163_E1 | Le_N7_S163 | 62044 | 62225 | 182 | GGAAGCGAACAAACAAGCAT | GTCGTTGGCCGAAAAGTTTA | InDel | 0.590 | 0.515 | 2.440 |
| S299_E1 | Le_N7_S299 | 61941 | 62164 | 224 | TACTGTTTTTGCAGCGCATC | CGTCAACGGTTTCAAGATCA | InDel | 0.496 | 0.373 | 1.984 |
| S413_E1 | Le_N7_S413 | 102632 | 102787 | 156 | CGCCATCAAGAGGGAAATTA | GACGCGTCGCCTATATTGAT | InDel | 0.438 | 0.342 | 1.779 |
| S81_E1 | Le_N7_S81 | 85127 | 85330 | 204 | CCTGGAGGGACAAATGAGAA | CCACGATCCTGAGACCAAAT | InDel | 0.414 | 0.328 | 1.705 |
| S90_E1 | Le_N7_S90 | 66151 | 66392 | 242 | TTCGACGACTCAACGATGTC | GAGGAATTGAGGAGCAGACG | InDel | 0.378 | 0.306 | 1.607 |
| S278_ID1 | Le_N7_S278 | 62317 | 62606 | 290 | TGGCTCGCTTTTCTTCTGAT | CGGCGGTAGTACCACAGAGT | InDel | 0.382 | 0.348 | 1.619 |
| S278_ID3 | Le_N7_S278 | 26952 | 27189 | 238 | ATTGCGATGCAGAAAATTCC | GAGAAAGCATCCTCCACGAG | InDel | 0.492 | 0.433 | 1.968 |
| S278_ID4 | Le_N7_S278 | 87556 | 87853 | 298 | TGGATATGAGGATTGCCACA | ATTTGAGCATGGAGCGAGAT | InDel | 0.492 | 0.431 | 1.968 |
| S278_ID10 | Le_N7_S278 | 32629 | 32884 | 256 | GGCTTTCCGAAAACGTATCA | GGTCTCTCGATCATCACCGT | InDel | 0.483 | 0.367 | 1.936 |
| S278_ID9 | Le_N7_S278 | 116481 | 116744 | 264 | CGCTATCTACCTACGCACCC | AAAGCGCAAAAGATGGTGTC | InDel | 0.668 | 0.595 | 3.008 |
| S278_ID12 | Le_N7_S278 | 64206 | 64428 | 223 | ACCTACAACCGTGCACAACA | CGGATAGCTTGGAAGGAGGT | InDel | 0.444 | 0.346 | 1.800 |
| S278_ID14 | Le_N7_S278 | 103140 | 103385 | 246 | CGCTCAGCGATAATTTTGGT | CCACCGATTGCCTTGTAGAT | InDel | 0.461 | 0.354 | 1.854 |
| S278_ID16 | Le_N7_S278 | 55799 | 56023 | 225 | ACGCACACCTCCACCAGTAT | ATGACAACGAGAGGGTACGG | InDel | 0.697 | 0.638 | 3.301 |
| S278_ID20 | Le_N7_S278 | 122445 | 122690 | 246 | TGACGGAGCAGACAGTTGAC | AGGATTATCCAGGACGCCTT | InDel | 0.660 | 0.590 | 2.942 |
| S278_ID33 | Le_N7_S278 | 136219 | 136451 | 233 | TACTGCGCAAAAGTACACCG | ACGCGAAATACTAGGCTGGA | InDel | 0.389 | 0.313 | 1.636 |
| S278_ID28 | Le_N7_S278 | 128632 | 128886 | 255 | TCCAGCAGTTCATTCAGCAG | GAAAAATATGTTTGCGCGGT | InDel | 0.632 | 0.556 | 2.719 |
| S278_ID32 | Le_N7_S278 | 142546 | 142824 | 279 | TACCTTCCAAAGCTGTCGCT | CTGCAATTGCGTTAGGACAA | InDel | 0.464 | 0.356 | 1.864 |
| S278_ID34 | Le_N7_S278 | 85659 | 85929 | 271 | ATTCATTCAGCCACCGTTTC | AGGTCGGGAAGAAGAAGCTC | InDel | 0.533 | 0.439 | 2.142 |
| S278_ID36 | Le_N7_S278 | 44448 | 44629 | 182 | GAGCAGGCAGAACTTTCCAC | TTCGTAGGACGAGAACGCTT | InDel | 0.500 | 0.375 | 2.000 |
| S278_ID37 | Le_N7_S278 | 62350 | 62625 | 276 | TTGTTCTTGCAAGCCAATGA | CAGCCTGAGGAAGAATTTGC | InDel | 0.542 | 0.457 | 2.186 |
| S278_ID41 | Le_N7_S278 | 119722 | 119936 | 215 | TAGCTCGGCCTGTGATAGGT | TGGTCTCCCTCCTCAATCTG | InDel | 0.500 | 0.375 | 2.000 |
| S278_F/R | Le_N7_S278 | 128796 | 129016 | 221 | GGGATTGTTCATGGTTGGAC | GGGCGTTGAATAGTCATGGT | InDel | 0.499 | 0.374 | 1.996 |
| S542_ID1 | Le_N7_S542 | 13136 | 13359 | 224 | GTGAGTATTGTGGGCGGACT | TTGCTCTGCTCATTGGTTTG | InDel | 0.645 | 0.574 | 2.816 |
| S412_ID1 | Le_N7_S412 | 23933 | 24228 | 296 | AGACTCCAGTACGCGACGTT | GAAAGAATCCGGAGTGACGA | InDel | 0.265 | 0.230 | 1.361 |
| S108_ID1 | Le_N7_S108 | 4050 | 4295 | 246 | TGATGATGGAGAAACACAAACC | ACCTGTTGCACAGGGGTATC | InDel | 0.622 | 0.554 | 2.648 |
| S260_ID1 | Le_N7_S260 | 69336 | 69602 | 267 | ATTCGAACGACCCGTATCAG | ACGTTGAAACCAACCGCTAC | InDel | 0.627 | 0.551 | 2.682 |
| S476_ID1 | Le_N7_S476 | 194089 | 194386 | 298 | AGTGAGCGGCTACGAGTCAT | ATTTTGTGCAACAGCAGCAG | InDel | 0.661 | 0.610 | 2.950 |
| S603_ID1 | Le_N7_S603 | 126930 | 127208 | 279 | CAGTGTTATGATGTTCGCCG | GGCGTGTATGGTCGTCTTTT | InDel | 0.500 | 0.380 | 1.999 |
| S267_ID1 | Le_N7_S267 | 14511 | 14800 | 290 | TTTTCCCTCCAATGAAGTCG | TTTTCGACATTCATCCAGCA | InDel | 0.500 | 0.380 | 1.999 |
| S649_ID1 | Le_N7_S649 | 35018 | 35271 | 254 | TGGGTCTGGAGAACGACTTC | ACTCCAGAATTCCATCGTCG | InDel | 0.454 | 0.361 | 1.832 |
| S255_ID1 | Le_N7_S255 | 22691 | 22987 | 297 | GTCGGAGATTCGTCCACAGT | ATCTTCATGGACGCGAAAAG | InDel | 0.523 | 0.453 | 2.097 |
| S18_ID1 | Le_N7_S18 | 2905 | 3196 | 292 | TTTTATCACCAAAGCTGGGC | CCACAATCACAAACTGGTCG | InDel | 0.342 | 0.284 | 1.520 |
| S26_ID1 | Le_N7_S26 | 108998 | 109248 | 251 | CGAGAGTCGAAAGGATCTGG | ACGTTTGACAAGATCCGAGG | InDel | 0.680 | 0.631 | 3.125 |
| S32_ID1 | Le_N7_S32 | 23113 | 23369 | 257 | GCAGCGAGAACTTGGAAAAA | TGCAGACAAAGGCTGACAAG | InDel | 0.217 | 0.193 | 1.277 |
| S44_ID1 | Le_N7_S44 | 113701 | 113993 | 293 | CTTCGTCGTGACGTCTTTCA | GCGGAAAGTAGTGGCAAAGT | InDel | 0.688 | 0.630 | 3.205 |
| S46_ID1 | Le_N7_S46 | 10118 | 10261 | 144 | ACCGAAATCCGTTAGCAATG | CTGGTACTGGTGGGGAAGAA | InDel | 0.309 | 0.261 | 1.447 |
| S48_ID1 | Le_N7_S48 | 17029 | 17299 | 271 | GATCGAAAATGTGGGATGCT | AAAGCCAAGGGGTTGAAGTT | InDel | 0.380 | 0.347 | 1.612 |
| S56_ID1 | Le_N7_S56 | 20485 | 20745 | 261 | CGTCTACGTCCAAACACCCT | TGTGTGCTCCATTGGGTAAA | InDel | 0.316 | 0.266 | 1.462 |
| S66_ID1 | Le_N7_S66 | 61686 | 61910 | 225 | TAAAGGCTGAGACCGCTGAT | CATTTTCCTTTTTGGTGCGT | InDel | 0.491 | 0.391 | 1.965 |
| S72_ID1 | Le_N7_S72 | 7177 | 7440 | 264 | CTGCGGTTGAGGAAGAAGAC | AAGGTCAGATCGAGGCAGAA | InDel | 0.284 | 0.249 | 1.396 |
| S75_ID1 | Le_N7_S75 | 43997 | 44145 | 149 | TGCTTCTCCGTCAAATACCC | CACTGCACCCTACAACCCTT | InDel | 0.502 | 0.397 | 2.006 |
| S78_ID1 | Le_N7_S78 | 10478 | 10666 | 189 | AGCGGACCGTAGAAGATTCA | GGAAGAAGCAGCAAAAGTGG | InDel | 0.488 | 0.437 | 1.955 |
| S81_ID1 | Le_N7_S81 | 65214 | 65512 | 299 | TAAGTATTCTGATGCGGGGC | GCGCGAACTATTACAGAGGC | InDel | 0.566 | 0.506 | 2.302 |
| S83_ID1 | Le_N7_S83 | 86197 | 86414 | 218 | GCTTTCCATCACCGTCCTTA | CTGAAGGTGAGCACCGTTTT | InDel | 0.491 | 0.370 | 1.964 |
| S100_ID1 | Le_N7_S100 | 98716 | 98952 | 237 | ATCCGCTAGCTAACGACGAA | GTTACAACCGACGCAGGTCT | InDel | 0.218 | 0.197 | 1.279 |
| S126_ID1 | Le_N7_S126 | 114581 | 114843 | 263 | GAGGTTCGGGATGAGTTTGA | TATGATGTTTTTCCACCGCA | InDel | 0.494 | 0.372 | 1.975 |
| S131_ID1 | Le_N7_S131 | 136840 | 137119 | 280 | GCAGCAAGTCCTCTGAAACC | CCGCTCCATCTGATTTTGTT | InDel | 0.526 | 0.419 | 2.108 |
| S147_ID1 | Le_N7_S147 | 22213 | 22454 | 242 | CGACTGAAAAGAGAGTGGGC | GCGTAATTTTTCCGAGTGGA | InDel | 0.280 | 0.241 | 1.389 |
| S160_ID1 | Le_N7_S160 | 11033 | 11269 | 237 | AGATCCGAACGAGCGTCTAA | CGTGTCCCTTTCCTGTTGTT | InDel | 0.348 | 0.288 | 1.535 |
| S163_ID1 | Le_N7_S163 | 9216 | 9390 | 175 | AACTGCCCGATGCAGTCTAC | TATGCGCGCAGTGTGAAATA | InDel | 0.236 | 0.208 | 1.308 |
| S178_ID1 | Le_N7_S178 | 80686 | 80963 | 278 | GGAAGACCTGTGCGGTATGT | TATGGCAATTTGGGAGGAAC | InDel | 0.400 | 0.320 | 1.665 |
| S180_ID1 | Le_N7_S180 | 115899 | 116146 | 248 | GAAGGGTGGACTCTGGTTGA | AAAGCGGGGAAGAGAAATGT | InDel | 0.615 | 0.542 | 2.597 |
| S183_ID1 | Le_N7_S183 | 9128 | 9426 | 299 | GTCGGCATCGTATCTGGTTT | AGACCGTCCTGATCCTCCTT | InDel | 0.254 | 0.222 | 1.341 |
| S205_ID1 | Le_N7_S205 | 47048 | 47317 | 270 | GAACCTCCCGATCCAGATTT | CGGGAAACGGTTCATACAAT | InDel | 0.409 | 0.325 | 1.692 |
| S143_ID1 | Le_N7_S143 | 51993 | 52279 | 287 | GCGTCCACTTGCGAAATTAT | ATATCATCTCCGGGCTGTTG | InDel | 0.491 | 0.370 | 1.964 |
| S222_ID1 | Le_N7_S222 | 112662 | 112903 | 242 | ATACCAACCTTCCCACCCTC | CGCAAATTGTGACTCGAAAA | InDel | 0.626 | 0.564 | 2.673 |
| S235_ID1 | Le_N7_S235 | 99877 | 100176 | 300 | ACTGCCAACATTGCCTCTCT | CCACCTGGGTTGTTCTCTGT | InDel | 0.500 | 0.375 | 2.000 |
| S240_ID1 | Le_N7_S240 | 131137 | 131387 | 251 | CTTCATGGGCAACTGGTTTT | GCACACGTAAAACGTAGCGA | InDel | 0.618 | 0.552 | 2.616 |
| S244_ID1 | Le_N7_S244 | 23077 | 23345 | 269 | TCGCAGGAAAAGGGACTAAG | CTTGGATACTTCGCCTCTCG | InDel | 0.498 | 0.374 | 1.991 |
| S259_ID1 | Le_N7_S259 | 1551 | 1768 | 218 | CAAGCACTTGGCAAGATCAG | GGGGAGACTCAACCTCACAA | InDel | 0.372 | 0.310 | 1.591 |
| S274_ID1 | Le_N7_S274 | 2811 | 3052 | 242 | ATTGTCGAAACCGTCTGTCC | AGGTAAACCATGGAGGTCCC | InDel | 0.383 | 0.310 | 1.622 |
| S306_ID1 | Le_N7_S306 | 37635 | 37911 | 277 | TCATCGATTGACGGTTTTGA | ATGGATTGCGAGAATTGACC | InDel | 0.268 | 0.238 | 1.367 |
| S314_ID1 | Le_N7_S314 | 44661 | 44893 | 233 | CTGCTGAGGAGCGTTAGCTT | TGAGGCGCATTCAAGTATGA | InDel | 0.556 | 0.484 | 2.253 |
| S322_ID1 | Le_N7_S322 | 42624 | 42857 | 234 | ACCGGCGATATACAAACAGG | GCGTCCACAAAGGAATCTGT | InDel | 0.498 | 0.374 | 1.994 |
| S323_ID1 | Le_N7_S323 | 1044 | 1303 | 260 | ATTTGGATGGTTCGCGTAAG | CGAAACTCCAGATGACAGCA | InDel | 0.329 | 0.275 | 1.491 |
| S333_ID1 | Le_N7_S333 | 33474 | 33683 | 210 | GTCCCTTTTTGACACCCAGA | ATTCGCTGAAGATGGAATGG | InDel | 0.418 | 0.373 | 1.718 |
| S337_ID1 | Le_N7_S337 | 22155 | 22328 | 174 | TGGGAGCCTGTAAAAATTGC | TGCGGCCATGATGATAGTTA | InDel | 0.605 | 0.522 | 2.534 |
| S338_ID1 | Le_N7_S338 | 26342 | 26613 | 272 | ATTCCCCACAGTTAGGGAGG | CGTACGATGCGTAAAACACG | InDel | 0.500 | 0.375 | 1.999 |
| S340_ID1 | Le_N7_S340 | 9023 | 9314 | 292 | ATTTTCCCTTCAAGCCGAAT | TTCTCGAGGGACTGACTCGT | InDel | 0.265 | 0.230 | 1.361 |
| S346_ID1 | Le_N7_S346 | 170688 | 170987 | 300 | GAGCGCTGGGATTCATAGAG | GAAGCCCACTCATCTTCGAC | InDel | 0.389 | 0.313 | 1.636 |
| S358_ID1 | Le_N7_S358 | 17391 | 17676 | 286 | TGTAGCCCCTTAAAACGCAC | GCTGATAGGAGCTGACTGGG | InDel | 0.499 | 0.404 | 1.996 |
| S363_ID1 | Le_N7_S363 | 65425 | 65592 | 168 | TCTCCACCATTCCTTTTTGG | TGTACCTGACGAAGGGGTTC | InDel | 0.434 | 0.340 | 1.766 |
| S384_ID1 | Le_N7_S384 | 13880 | 14131 | 252 | TTCTGCAGGAGGCAATTCTT | GAAGGTCCAGCCATTGAAAA | InDel | 0.315 | 0.275 | 1.460 |
| S392_ID1 | Le_N7_S392 | 41263 | 41450 | 188 | ACCCTCTACAGGGACCGTTT | ACCGCGTTTTTGATTTTCAC | InDel | 0.482 | 0.366 | 1.930 |
| S395_ID1 | Le_N7_S395 | 65502 | 65774 | 273 | CGGAGGTGTAACCTCGTCAT | AAAAGTTGACGAAGCAGGGA | InDel | 0.571 | 0.507 | 2.331 |
| S415_ID1 | Le_N7_S415 | 8135 | 8415 | 281 | AGGGTTGGAGGTCAACAGTG | ATACAGAACCGTACGGCGAG | InDel | 0.316 | 0.266 | 1.462 |
| S427_ID1 | Le_N7_S427 | 10090 | 10348 | 259 | CAAAATCGCAGCTTACACCA | GAAGGCACCAGCCAAAGTTA | InDel | 0.578 | 0.494 | 2.371 |
| S442_ID1 | Le_N7_S442 | 30722 | 30942 | 221 | GGCGTTGTATTGCTTCCAGT | CAGTGTGACGGACTCCAGAA | InDel | 0.375 | 0.309 | 1.600 |
| S444_ID1 | Le_N7_S444 | 37753 | 37974 | 222 | GCAACGGCGTGATAGTACCT | CTTTGAACGAAGGAAGACGC | InDel | 0.412 | 0.327 | 1.700 |
| S446_ID1 | Le_N7_S446 | 4976 | 5224 | 249 | GCAACATGAAGTGAGAGGCA | CAGAAGATGCGCATACTGGA | InDel | 0.426 | 0.340 | 1.742 |
| S379_ID1 | Le_N7_S379 | 45130 | 45358 | 229 | GCAGGTGGATCCCTCTGTTA | CGAGGACGGAGATGGATTTA | InDel | 0.280 | 0.241 | 1.389 |
| S449_ID1 | Le_N7_S449 | 33530 | 33794 | 265 | CATTGATCATGTTCCATCGC | TGGATAAGGTGGATTGGCAT | InDel | 0.282 | 0.248 | 1.392 |
| S457_ID1 | Le_N7_S457 | 34466 | 34699 | 234 | CGCTCAGGAGAAAACTGGTC | TTCTCCGGTCCATGAAAATC | InDel | 0.748 | 0.705 | 3.963 |
| S473_ID1 | Le_N7_S473 | 6272 | 6482 | 211 | TCACATCACTTGCTTCTGCC | GTCGCGTTATCAAAGCAACA | InDel | 0.485 | 0.368 | 1.944 |
| S480_ID1 | Le_N7_S480 | 23344 | 23571 | 228 | GCGCCGAGTTATAGGTGTGT | CAAAGTTATCCCTGCGAAGC | InDel | 0.517 | 0.464 | 2.071 |
| S488_ID1 | Le_N7_S488 | 58731 | 58970 | 240 | TTTCCATAGGCCTTTTGTCG | ATGACGGTGAAGTCTGACCC | InDel | 0.604 | 0.523 | 2.525 |
| S492_ID1 | Le_N7_S492 | 61241 | 61538 | 298 | AAGCGACTGCAAATCCAACT | GAGGACGAGGAAGACGACTG | InDel | 0.421 | 0.337 | 1.729 |
| S504_ID1 | Le_N7_S504 | 12752 | 12903 | 152 | AACAACGGACCGAAAAACAG | TCTGTTGGGGTGTGTGCTTA | InDel | 0.494 | 0.372 | 1.975 |
| S534_ID1 | Le_N7_S534 | 68169 | 68461 | 293 | ACTGCAGCGCTTTCGATTAC | TTGGACAGACCCATGCTACA | InDel | 0.552 | 0.477 | 2.230 |
| S535_ID1 | Le_N7_S535 | 32684 | 32862 | 179 | AGAATCCACAGGCATTTTGC | TCCAAGGCCCAGATACATTC | InDel | 0.377 | 0.318 | 1.606 |
| S560_ID1 | Le_N7_S560 | 24961 | 25159 | 199 | CATCCACCTTTGTTTTGCCT | CCGAATGCCGATACTTCCTA | InDel | 0.487 | 0.369 | 1.951 |
| S598_ID1 | S598_ID1 | 26132 | 26352 | 221 | CCGTGGTTCTCATTCCTTGT | GTTCAGCCAAGCAAGTCACA | InDel | 0.662 | 0.588 | 2.959 |
| S604_ID1 | Le_N7_S604 | 4855 | 5142 | 288 | GGCGAACACGTTCCTAAAAG | AAGCCAAAGGACTCATCGTG | InDel | 0.500 | 0.375 | 2.000 |
| S605_ID1 | Le_N7_S605 | 20380 | 20637 | 258 | ACGCTTGGGAACCTTATCCT | TAAACCAACAAACTTCCGCC | InDel | 0.424 | 0.343 | 1.736 |
| S653_ID1 | Le_N7_S653 | 40147 | 40368 | 222 | CGATAGCAATTCGAGCAACA | CGCCTTTCACGCTTCTACTT | InDel | 0.278 | 0.239 | 1.385 |
| S655_ID1 | Le_N7_S655 | 30734 | 30994 | 261 | CGGTGAAGCAGTTGTTGAGA | CGGTTGCTGTTCCGAGTTAT | InDel | 0.312 | 0.263 | 1.453 |
| S699_ID1 | Le_N7_S699 | 98981 | 99197 | 217 | TGACGATACTGGACTGCCTG | ATGCTGAAGATCGGGAGATG | InDel | 0.461 | 0.375 | 1.855 |
| S767_ID1 | Le_N7_S767 | 23431 | 23718 | 288 | TGCTTGCAGTATAGGACCCC | TCCTTGAGGCAAGCAGATTT | InDel | 0.483 | 0.366 | 1.935 |
| S103_SSR1 | Le_N7_S103 | 24902 | 24919 | 234 | CGAACTCGACGTACCCTCTC | CAATCATCTGTCGTGAACCG | SSR | 0.429 | 0.369 | 1.751 |
| S321_SSR1 | Le_N7_S321 | 33372 | 33389 | 226 | TCGATAATACCCCAACCCAA | CAATTGAACATAACCGTGCG | SSR | 0.372 | 0.310 | 1.591 |
| S124_ID5 | Le_N7_S124 | 74331 | 74621 | 291 | CGCCAAATATGAGCTTCCAT | TGGCTGGTCAGTGTCTTCAG | InDel | 0.666 | 0.593 | 2.990 |
| S127_ID5 | Le_N7_S127 | 25037 | 25281 | 245 | AAGAAACTGAACGCAAGGGA | TAGTACAGCCTTCCATCCCG | InDel | 0.672 | 0.611 | 3.044 |
| S129_ID5 | Le_N7_S129 | 120600 | 120890 | 291 | GGGATATGCGTAAGGGGTTT | TACCCACTTGCTTACCTGGG | InDel | 0.477 | 0.363 | 1.913 |
| S131_ID5 | Le_N7_S131 | 121874 | 122083 | 210 | AGATTGCTTGGAACAATGGC | ATGACCTGAAAACGGTGCTC | InDel | 0.118 | 0.114 | 1.134 |
| S152_ID5 | Le_N7_S152 | 6810 | 7080 | 271 | TTGAGTGTTGCAAGGAGCAG | TATAGCAGTTCCGGTGGTCC | InDel | 0.571 | 0.482 | 2.331 |
| S160_ID5 | Le_N7_S160 | 8313 | 8562 | 250 | TTATTTGGAAATCGCTTGGC | TTCCTAGGATCAAGATGGCG | InDel | 0.345 | 0.285 | 1.527 |
| S180_ID5 | Le_N7_S180 | 110358 | 110647 | 290 | GCTCCTGTGAAGCCTGAGAC | ATCGGCACGAACCTATTCTG | InDel | 0.448 | 0.348 | 1.812 |
| S184_ID5 | Le_N7_S184 | 56585 | 56771 | 187 | GCACTATTTTCCGACAAGCC | TGTTGAGGAAGCAACCAGTG | InDel | 0.389 | 0.322 | 1.636 |
| S216_ID5 | Le_N7_S216 | 1554 | 1792 | 239 | CGCTCCGTGAGGATGATATT | GCTGATGGTAGGCCGATAAA | InDel | 0.268 | 0.232 | 1.365 |
| S225_ID5 | Le_N7_S225 | 57507 | 57804 | 298 | GGTGTGCAAAATCCGAAACT | CACTGTACCGCAGGCTATGA | InDel | 0.473 | 0.418 | 1.896 |
| S240_ID5 | Le_N7_S240 | 153011 | 153237 | 227 | CTCCCTACCATTCTCAAGCG | GTGATGTCGTGATGAATCGG | InDel | 0.434 | 0.349 | 1.766 |
| S243_ID5 | Le_N7_S243 | 176042 | 176317 | 276 | GATGCAGGATTGGGAGAAGA | CGAACATGTGATGCGATACC | InDel | 0.533 | 0.452 | 2.141 |
| S244_ID5 | Le_N7_S244 | 7655 | 7920 | 266 | TTGAGTGTAGTTAGGCGGGG | TTTGAGATGCGGCAGTGTAG | InDel | 0.475 | 0.372 | 1.904 |
| S247_ID5 | Le_N7_S247 | 50979 | 51275 | 297 | TGTTGCGATGAGCACTTTTC | CCTCTTCCGCATTGGAATTA | InDel | 0.262 | 0.227 | 1.354 |
| S253_ID5 | Le_N7_S253 | 355 | 631 | 277 | GGACTACCGGACGCTTGTTA | CATGTTCTGCTTGCCTTTGA | InDel | 0.321 | 0.273 | 1.472 |
| S254_ID5 | Le_N7_S254 | 206257 | 206517 | 261 | CATTGCCGCCTTTTAGAGAG | GAATATGCATACACGTGGCG | InDel | 0.316 | 0.291 | 1.462 |
| S257_ID5 | Le_N7_S257 | 103731 | 103988 | 258 | GAAATTTGATGGTCTCCCCA | CGGAAAAGGGTAGAAAAGCC | InDel | 0.363 | 0.320 | 1.570 |
| S258_ID5_2 | Le_N7_S258 | 68406 | 68622 | 217 | TAAACGGCATGGTCCAAGAT | AAGGAGAGGCAGGATCACAA | InDel | 0.501 | 0.405 | 2.004 |
| S258_ID5 | Le_N7_S258 | 47212 | 47463 | 252 | GTGTAAAGTCGGGCGCTTAG | CACTGCCAGGTTCATAGGGT | InDel | 0.282 | 0.245 | 1.393 |
| S259_ID5 | Le_N7_S259 | 16333 | 16613 | 281 | AATGGTCGATCGGACTTGAG | GAAAGGGATGTTGGACGAAG | InDel | 0.614 | 0.535 | 2.594 |
| S26_ID5 | Le_N7_S26 | 63452 | 63682 | 231 | GGAAAATGCGCATCAGAAAT | GATGTGCCCGTCATTTTCTT | InDel | 0.447 | 0.347 | 1.808 |
| S273_ID5 | Le_N7_S273 | 59931 | 60173 | 243 | TGGACTGCGTTACAGTTTGC | GACAGTGAACTTGGGGGAAA | InDel | 0.370 | 0.333 | 1.587 |
| S297_ID5 | Le_N7_S297 | 58956 | 59250 | 295 | CTCGGTATTGATGGGGAGAA | TGATCAGGAACACTCAAGCG | InDel | 0.309 | 0.261 | 1.447 |
| S31_ID5 | Le_N7_S31 | 57784 | 58054 | 271 | CAGTCCTCTTCTCCCAGTCG | TTTCAATAACCCCGACAAGC | InDel | 0.475 | 0.362 | 1.904 |
| S320_ID5 | Le_N7_S320 | 28347 | 28606 | 260 | TTTGTCTAGCCCGAGATGCT | GCTGTCAACTACCGCCATTT | InDel | 0.628 | 0.555 | 2.687 |
| S324_ID5 | Le_N7_S324 | 5874 | 6105 | 232 | GAACAGTCATTGGTTGCCCT | TTGATGTCGAACTGCCTACG | InDel | 0.474 | 0.367 | 1.900 |
| S328_ID5 | Le_N7_S328 | 9984 | 10213 | 230 | TCCATGACCGATCAACAGAA | AGGAAGGAACCGTCCAAACT | InDel | 0.480 | 0.365 | 1.921 |
| S333_ID5 | Le_N7_S333 | 34127 | 34313 | 187 | AAACTCTTTTCACCATGCCG | CACTGGTTCCACAACAATGC | InDel | 0.488 | 0.437 | 1.953 |
| S381_ID5 | Le_N7_S381 | 27953 | 28138 | 186 | AGGGAGAACACGAGCAGAAA | CCCATCTTTTGTGTCCGATT | InDel | 0.438 | 0.351 | 1.780 |
| S388_ID5 | Le_N7_S388 | 176154 | 176440 | 287 | CGAGGTTGGTAGTGCTGACA | AGAGCTCGAAATCCAGGACA | InDel | 0.467 | 0.358 | 1.875 |
| S438_ID5 | Le_N7_S438 | 50777 | 51030 | 254 | GCTAGGCCCGATATCACTCA | ATGGTATCCGCGAGAATCAG | InDel | 0.399 | 0.319 | 1.664 |
| S446_ID5 | Le_N7_S446 | 38081 | 38373 | 293 | TTCAGGTCAACCGTTGTGAA | CGCTCATTGGGTTATGGAAT | InDel | 0.725 | 0.675 | 3.633 |
| S46_ID5 | Le_N7_S46 | 12231 | 12502 | 272 | GGAAACATCTTTCAATCGGC | TCGTGGAAACGACACAAAAA | InDel | 0.329 | 0.275 | 1.491 |
| S476_ID5 | Le_N7_S476 | 11773 | 12057 | 285 | TGCAAGACAACCGTTGGTTA | AGTGTTGACCTGGAAATGGG | InDel | 0.391 | 0.361 | 1.641 |
| S484_ID5 | Le_N7_S484 | 18209 | 18498 | 290 | ATGCCTGCACCTCATCTACC | GTATCTCAGCGCCAAACCAT | InDel | 0.238 | 0.210 | 1.312 |
| S491_ID5 | Le_N7_S491 | 22156 | 22433 | 278 | TAGGATGTCGACCTCAAGGG | TTGCCATTCACAAAACCAGA | InDel | 0.423 | 0.333 | 1.732 |
| S492_ID5 | Le_N7_S492 | 73187 | 73464 | 278 | AATGATTTGTCGGTTCTCCG | CCACCGGAGGCTACAGAGTA | InDel | 0.511 | 0.391 | 2.045 |
| S499_ID5 | Le_N7_S499 | 252012 | 252235 | 224 | CGGGGTGAGTATCTCTTGGA | GTGATGGCCGCTAACAATCT | InDel | 0.457 | 0.407 | 1.841 |
| S51_ID5 | Le_N7_S51 | 26316 | 26576 | 261 | TTGAGGATCACTGGATGTCG | TTCCAATCATAGCCGATTCC | InDel | 0.362 | 0.315 | 1.568 |
| S53_ID5 | Le_N7_S53 | 615667 | 615939 | 273 | GAGCTTGGGGAACTTGAACA | TTGAGAGGCAAGGCTTCTGT | InDel | 0.529 | 0.480 | 2.124 |
| S530_ID5 | Le_N7_S530 | 1521 | 1708 | 188 | CGTCAGCCTCGTCTACTTCC | GGGAACAAAAACCATCGAAA | InDel | 0.246 | 0.216 | 1.326 |
| S553_ID5 | Le_N7_S553 | 15272 | 15500 | 229 | TCCGCACTTCATTAGCTCCT | TCCACCATCACACTCGGTAA | InDel | 0.383 | 0.310 | 1.622 |
| S603_ID5 | Le_N7_S603 | 98065 | 98361 | 297 | TTTGCTTTCAGCGAGTTTCA | ATCCGGGGAGTAGCAAAGAT | InDel | 0.336 | 0.279 | 1.506 |
| S60_ID5 | Le_N7_S60 | 41508 | 41784 | 277 | TCTGTCGCACCTTCACTCAG | GATCGCCTTTCCTCGTTGTA | InDel | 0.622 | 0.552 | 2.645 |
| S649_ID5 | Le_N7_S649 | 50001 | 50233 | 233 | TTTCAAACCGATGTCGTGAA | CGACGCTCGATATTCCATTT | InDel | 0.475 | 0.362 | 1.904 |
| S66_ID5 | Le_N7_S66 | 29884 | 30142 | 259 | AAAACCCAGTCCGGAAAAAC | CACGGTTTTCATTGTGCAAC | InDel | 0.612 | 0.536 | 2.576 |
| S723_ID5 | Le_N7_S723 | 29837 | 29976 | 140 | TGCATCCTGCAAAGAACAAG | TCTCTTCGTTTCCTTTGGGA | InDel | 0.334 | 0.282 | 1.502 |
| S72_ID5 | Le_N7_S72 | 20392 | 20691 | 300 | GATCGCAGCTTGGAGCTAGT | AACAGAGAACCAGCGAAGTGA | InDel | 0.389 | 0.313 | 1.636 |
| S767_ID5 | Le_N7_S767 | 26309 | 26570 | 262 | AAAAGCAAGTCCTCGGTTGA | TATCTCGAGAAGTGCGCAGA | InDel | 0.417 | 0.334 | 1.715 |
| S88_ID5 | Le_N7_S88 | 40692 | 40953 | 262 | ATTTCGATGCGGAAATTGAC | TTGAATATCGACATGGGGGT | InDel | 0.561 | 0.463 | 2.279 |
| S95_ID5 | Le_N7_S95 | 20356 | 20607 | 252 | GACGATCGCTTTTTGCTGTT | CGAATTTGATCGTCGGAAGT | InDel | 0.768 | 0.731 | 4.309 |
| S641_SSR1 | Le_N7_S641 | 88544 | 88803 | 260 | GAGAGGAAAACGGAAAAGGG | GGTTTATCTCGTGGCGTGTT | SSR | 0.579 | 0.527 | 2.375 |
| S265_SSR1 | Le_N7_S265 | 105625 | 105805 | 181 | ATTCCAAGGCCAAACTGTTG | CGAATTTTCCCGTCGTTAGA | SSR | 0.490 | 0.381 | 1.962 |
| S76_ID1 | Le_N7_S76 | 38904 | 39109 | 206 | GTGGTTGCAGCTTCTTAGGC | TAGGTGAAGTTCCGAGCGAT | InDel | 0.597 | 0.519 | 2.483 |
| S759_ID1 | Le_N7_S759 | 49133 | 49349 | 217 | GCGATCCTGTGTTCATGTTG | GAGCACTCAGCAAAGGAAGC | InDel | 0.511 | 0.391 | 2.045 |
| S730_ID1 | Le_N7_S730 | 19978 | 20240 | 263 | AGCGGATCAGCTACACACAG | GGTCTATACGCTGGCTCAGG | InDel | 0.568 | 0.493 | 2.314 |
| S710_ID1 | Le_N7_S710 | 25203 | 25489 | 287 | GTTCGTCGGATGAAGGAGAG | GGAAGCACCAGGACATTGTT | InDel | 0.577 | 0.488 | 2.363 |
| S700_ID1 | Le_N7_S700 | 164283 | 164437 | 155 | CTAAAACCCGATTCACCGAA | TCACAGACGATGAGAGTGGC | InDel | 0.435 | 0.341 | 1.771 |
| S647_ID1 | Le_N7_S647 | 11133 | 11330 | 198 | ATTTCGCCTTGGGTTTCTTT | AACACCTTCTGCACCTCCAC | InDel | 0.268 | 0.232 | 1.365 |
| S621_ID1 | Le_N7_S621 | 71242 | 71417 | 176 | CGAGCAACGAAACAAGTTGA | AGGGGGTCCTGAGTGACTTT | InDel | 0.601 | 0.518 | 2.509 |
| S615_ID1 | Le_N7_S615 | 56000 | 56156 | 157 | ACCCTTTGGTGCCTTTCTTT | GGTGAAGGCGATAATGCTGT | InDel | 0.480 | 0.365 | 1.921 |
| S596_ID1 | S596_ID1 | 27300 | 27517 | 218 | CTGGGAGCTTCTGAGAATGG | CATCTGGCTTCATTACCCGT | InDel | 0.219 | 0.195 | 1.280 |
| S583_ID1 | S583_ID1 | 36588 | 36865 | 278 | CGGATCGGAATTCTCTTCAA | TGGTAGTTTTTGCGACCTCC | InDel | 0.656 | 0.582 | 2.906 |
| S548_ID1 | Le_N7_S548 | 13088 | 13384 | 297 | TTCGCCACCCTCTGAATATC | GTCCGCAACCAATTTCAAGT | InDel | 0.488 | 0.380 | 1.954 |
| S52_ID1 | Le_N7_S52 | 18486 | 18753 | 268 | CATTTTACTGAAGGCACGCA | TACCGCAATTCTTTCCGAAC | InDel | 0.500 | 0.375 | 1.999 |
| S500_ID1 | Le_N7_S500 | 4544 | 4782 | 239 | TTGATCACTCTCATTGCTCCA | TGTGACTGTGACAGGTGGGT | InDel | 0.638 | 0.566 | 2.761 |
| S470_ID1 | Le_N7_S470 | 174635 | 174892 | 258 | GCGCAAAGGCTATTCAAGAC | CAACGGAAACATCGGAAAGT | InDel | 0.399 | 0.319 | 1.664 |
| S400_ID1 | Le_N7_S400 | 4944 | 5240 | 297 | AACTCGTACGTCAAGGACGG | CTGATCAGCAAGGTCAACGA | InDel | 0.469 | 0.359 | 1.885 |
| S396_ID1 | Le_N7_S396 | 4050 | 4347 | 298 | AGTAGCTGGTGGAGAAGGCA | CGTGCCCACTACTAAGCCTC | InDel | 0.419 | 0.331 | 1.721 |
| S390_ID1 | Le_N7_S390 | 42551 | 42840 | 290 | CTGTGACGAACGCAACATCT | CCGATGGTAAATCATCCCAG | InDel | 0.292 | 0.269 | 1.413 |
| S36_ID1 | Le_N7_S36 | 23728 | 23988 | 261 | TCGGATGTAGTCCAAGTCTGG | GGGTAGCAGCCAGACCAATA | InDel | 0.496 | 0.373 | 1.984 |
| S336_ID1 | Le_N7_S336 | 83911 | 84178 | 268 | GGTTCACGAAGTGCAGAACA | CCAGTCACTAAATGGCGGTT | InDel | 0.391 | 0.334 | 1.642 |
| S335_ID1 | Le_N7_S335 | 20467 | 20754 | 288 | TTCTTTCCTTTGGCCCTTTT | TTGATTGATTGCACCGGATA | InDel | 0.477 | 0.363 | 1.913 |
| S325_ID1 | Le_N7_S325 | 133080 | 133353 | 274 | GGTCCTCAATCCGTAAACGA | AGATCGACGCAAACGGTATC | InDel | 0.332 | 0.280 | 1.496 |
| S29_ID1 | Le_N7_S29 | 32369 | 32631 | 263 | ATTCCGGAGTTGGAGGAGTT | CTGGTTAATTGTGTGGCGTG | InDel | 0.662 | 0.588 | 2.958 |
| S277_ID1 | Le_N7_S277 | 49710 | 49998 | 289 | TCCGTAGAAATGTAAGCGGC | CAGCTGCTCAGAATCGAATG | InDel | 0.686 | 0.628 | 3.186 |
| S231_ID1 | Le_N7_S231 | 181253 | 181478 | 226 | ACGTGTGGGGAAAATATTGG | GAGCGAGACGCTGAAGTACC | InDel | 0.398 | 0.350 | 1.662 |
| S211_ID1 | Le_N7_S211 | 64552 | 64799 | 248 | TCGGGCTGAGCTTGAGTTAT | AATGCTTGTCCTCCATCGTC | InDel | 0.704 | 0.647 | 3.374 |
| S206_ID1 | Le_N7_S206 | 30787 | 30962 | 176 | GAGATGCAGTGCCAGTTTCA | ACTCCCAACTCCCAGGCTAT | InDel | 0.647 | 0.574 | 2.831 |
| S192_ID1 | Le_N7_S192 | 54561 | 54845 | 285 | AAAACCAACGGGAGGAAAGT | TTCCCATGCTGACTAGGTCC | InDel | 0.410 | 0.326 | 1.694 |
| S191_ID1 | Le_N7_S191 | 60474 | 60651 | 178 | CGTAGTGGCGAAGGTGATTT | TGAAGTTCACCTGCTGCTGT | InDel | 0.257 | 0.224 | 1.345 |
| S755_ID5 | Le_N7_S755 | 54718 | 55009 | 292 | CGTTGATGGAAGATGCAAGA | GAACGCATATACGGCCAGTT | InDel | 0.397 | 0.351 | 1.658 |
| S140_ID5 | Le_N7_S140 | 124018 | 124299 | 282 | TCAGCGTGTAAAACCCTTCC | GGTTCACCACGTTTCTTCGT | InDel | 0.481 | 0.371 | 1.927 |
| S181_ID1 | Le_N7_S181 | 10573 | 10805 | 233 | GGTGGCGACTAGAATTGGAA | AAGCCATCAGCCTTTTTGAA | InDel | 0.506 | 0.383 | 2.023 |
| S171_ID1 | Le_N7_S171 | 26027 | 26255 | 229 | TTGCTGAGCTTTCGGTAGGT | GATCCAAAACCAAACGGCTA | InDel | 0.626 | 0.550 | 2.672 |
| S149_ID1 | Le_N7_S149 | 62090 | 62311 | 222 | ATCCACAATCAGCGGCTTAC | GCATTAGCAACAGTGGGGTT | InDel | 0.457 | 0.353 | 1.843 |
| S134_ID1 | Le_N7_S134 | 74233 | 74417 | 185 | GGGGCTGTGTGAATAGGAGA | GCAGTATCAAGGAAGGGTGC | InDel | 0.593 | 0.559 | 2.459 |
| S127_ID1 | Le_N7_S121 | 27159 | 27308 | 150 | GTCGAGATGCTAGACTCGGG | CACATACGATATGCATCGGC | InDel | 0.658 | 0.594 | 2.926 |
| S119_ID1 | Le_N7_S119 | 38857 | 39088 | 232 | GTTGAGGTTGCCAATGTTGA | TCAGTGTCGTTGTTGGGAGA | InDel | 0.475 | 0.368 | 1.905 |
| S115_ID1 | Le_N7_S115 | 15891 | 16178 | 288 | CGGTGGAGAAGTAGCAGAGC | GGCAGTTTCCTTAGTCGCAG | InDel | 0.323 | 0.271 | 1.476 |
| S339_ID1 | Le_N7_S339 | 37158 | 37300 | 143 | TCACCTTTCCTCTCTGCGTT | GAGTGTTGCACTGGAAGCAA | InDel | 0.538 | 0.461 | 2.165 |
| S458_ID5 | Le_N7_S458 | 51977 | 52224 | 248 | TGCAAAAATGGCTGTCTCAG | AGTCCGTCAAAACTGCCATC | InDel | 0.617 | 0.547 | 2.612 |
| S517_ID5 | Le_N7_S517 | 2229 | 2412 | 184 | AACGGCGTTAGTGGTAGTGG | GAGGTGGGATAACACTCGGA | InDel | 0.600 | 0.538 | 2.503 |
| S79_ID5 | Le_N7_S79 | 27114 | 27402 | 289 | AACTGCTGGCATTTGCTTTT | AATTCTCCTCCTCCCCTTCA | InDel | 0.298 | 0.253 | 1.424 |
| S539_ID1 | Le_N7_S539 | 12980 | 13198 | 219 | GCGTTATTTTGGTGCTTCGT | TTACCAACCCGCTACTTTGC | InDel | 0.627 | 0.554 | 2.685 |
| S35_inID1 | Le_N7_S35 | 1858 | 2096 | 239 | TCTTCGGGTTCAAGAGCAGT | ACCTCCGAGTGTCGGAATTA | InDel | 0.600 | 0.520 | 2.502 |
| S59_inID1 | Le_N7_S59 | 9560 | 9883 | 324 | GGTGGGCTTCATAAGCAGAA | AGCTCCAGAATTTCGCATGT | InDel | 0.435 | 0.341 | 1.771 |
| S73_inID1 | Le_N7_S73 | 79214 | 79511 | 298 | GGGGTAAATGAAGAAGGGGA | TCAACGTGCGTTTGATGTTT | InDel | 0.473 | 0.381 | 1.896 |
| S82_inID1 | Le_N7_S82 | 80354 | 80689 | 336 | AAAATCGAGCACTGGTTTGG | GATACATGGTACGGGGATGC | InDel | 0.334 | 0.285 | 1.501 |
| S93_inID1 | Le_N7_S93 | 4026 | 4314 | 289 | TTGGAGCCTTGAGGTTGAGT | ACGATGATGATGGAATGGGT | InDel | 0.443 | 0.345 | 1.796 |
| S106_inID1 | Le_N7_S106 | 29057 | 29367 | 311 | TCCGTGACAAGTTACCCACA | TAACATCAGGCTGCTCATCG | InDel | 0.154 | 0.142 | 1.183 |
| S121_inID1 | Le_N7_S121 | 16930 | 17198 | 269 | AGTCAACCGTTCGTTTCGTC | GCCTGAAATTCCTGCTCTTG | InDel | 0.533 | 0.443 | 2.140 |
| S150_inID1 | Le_N7_S150 | 39974 | 40147 | 174 | CGCATACTCTTTCGACACCA | CTTAGAGGTGCGCTTTCCAC | InDel | 0.564 | 0.495 | 2.295 |
| S169_inID1 | Le_N7_S169 | 286365 | 286702 | 338 | AGCAGGCCACGAGGTATATG | GACGGACGGAAAGGAAGTTT | InDel | 0.298 | 0.253 | 1.424 |
| S176_inID1 | Le_N7_S176 | 24479 | 24620 | 142 | GCAGATCTGTACGCAGCTTG | GACGAAGGAGGAATCTGCTG | InDel | 0.578 | 0.490 | 2.371 |
| S187_inID1 | Le_N7_S187 | 2131 | 2373 | 243 | AAATGAATTGAAACGGCGAG | CGTGAGGAATGGGGAGACTA | InDel | 0.451 | 0.349 | 1.820 |
| S189_inID1 | Le_N7_S189 | 20811 | 21044 | 234 | CCAGTCCAATCCATACCCAC | AGAAGGATGTGTTGATCGGG | InDel | 0.478 | 0.374 | 1.914 |
| S204_inID1 | Le_N7_S204 | 83398 | 83728 | 331 | AAAGCTCAGGGACGATGAGA | GTTCCGAGTCACCGTCAAAT | InDel | 0.431 | 0.338 | 1.758 |
| S214_inID1 | Le_N7_S214 | 4147 | 4466 | 320 | CGGCGATGAGAAGAAATAGC | TTCGTGTAAACCCTTGGGAG | InDel | 0.288 | 0.246 | 1.404 |
| S241_inID1 | Le_N7_S241 | 33040 | 33312 | 273 | GTGTTGAAGGATCCGGAAGA | GAGAAGGTGCTACTGGTGCC | InDel | 0.409 | 0.325 | 1.692 |
| S248_inID1 | Le_N7_S248 | 27235 | 27462 | 228 | TAAGAAGTTGGCGGGTGTTC | TTGTTCGAGGTCAATCCTCC | InDel | 0.634 | 0.558 | 2.733 |
| S266_inID1 | Le_N7_S266 | 6427 | 6704 | 278 | AACGTTGAAGGTTCGGATTG | TGACTTTCTCATGCTCGGTG | InDel | 0.499 | 0.375 | 1.998 |
| S298_inID1 | Le_N7_S298 | 74037 | 74317 | 281 | TAGGAGGCACGTTATCCTGG | AGCAGCAAGAGAGGAAGCAG | InDel | 0.617 | 0.543 | 2.609 |
| S319_inID1 | Le_N7_S319 | 58242 | 58512 | 271 | TAAACCCCACCGCACATATT | GCGCAGTAAATGGAGGGATA | InDel | 0.458 | 0.363 | 1.845 |
| S350_inID1 | Le_N7_S350 | 4721 | 5038 | 318 | TTCGAAGCATCTGTCATTGC | GATGGGTATGATCGCGAAGT | InDel | 0.459 | 0.380 | 1.849 |
| S383_inID1 | Le_N7_S383 | 3229 | 3445 | 217 | GTCTGAGGCCTTGAAGCAAC | TACTGTGATGGGTGGGACAA | InDel | 0.497 | 0.384 | 1.988 |
| S409_inID1 | Le_N7_S409 | 38189 | 38474 | 286 | AAGGGAAGGAAAAGGCGATA | AAAAGCCGGGGAATACAAAC | InDel | 0.238 | 0.215 | 1.313 |
| S416_inID1 | Le_N7_S416 | 10044 | 10383 | 340 | TCTACTCCCCGATTCACTGG | CCACTAGCCCCCTATTAGCC | InDel | 0.357 | 0.294 | 1.556 |
| S443_inID1 | Le_N7_S443 | 44580 | 44719 | 140 | GCTTGAATCCCTCAATTCCA | AACGAGTTTGCAGGAGCTGT | InDel | 0.164 | 0.150 | 1.196 |
| S469_inID1 | Le_N7_S469 | 1393 | 1678 | 286 | GGACTGGTTACGGTTCAGGA | TTGTCCATCGACATAAGCCA | InDel | 0.392 | 0.354 | 1.645 |
| S502_inID1 | Le_N7_S502 | 61586 | 61884 | 299 | GGCTCGTGGAGTGGTTGTAT | GTTGACCGGAAGGATTCTCA | InDel | 0.252 | 0.220 | 1.337 |
| S516_inID1 | Le_N7_S516 | 3149 | 3427 | 279 | GCTCGATAGCGCATCATGTA | CCGAAAGCTACAAGGATGGA | InDel | 0.443 | 0.402 | 1.797 |
| S724_inID1 | Le_N7_S724 | 3602 | 3917 | 316 | AGTCACGGACCACACTTTCC | TCTTTTCTTTCGGACAACCG | InDel | 0.392 | 0.319 | 1.644 |
| S704_inID1 | Le_N7_S704 | 82999 | 83145 | 147 | CTTTGCATCTTTGCTGGTCA | CACAAATCAAGGCGAAGGAT | InDel | 0.335 | 0.279 | 1.503 |
| S758_inID1 | Le_N7_S758 | 33074 | 33399 | 326 | CTCGCGGGTTGAAAGTGTAT | TGAAGTGGACAGTGAGCCAG | InDel | 0.467 | 0.358 | 1.875 |
| S766_inID1 | Le_N7_S766 | 13305 | 13533 | 229 | TACAAGTGCCAGCAGATTCG | ACAGCCTCAATGGGAGAAGA | InDel | 0.193 | 0.174 | 1.239 |
| S249_inID1 | Le_N7_S249 | 40317 | 40596 | 280 | ATTGGTTTCGACGAGAATGC | ATAAAGCTCGCTCGACGTGT | InDel | 0.241 | 0.212 | 1.318 |
| S536_inID1 | Le_N7_S536 | 17356 | 17502 | 147 | AGTAGGGACGGGATCGTTCT | GTGGGCTGATTTCGTTGACT | InDel | 0.244 | 0.220 | 1.324 |
| S551_inID1 | Le_N7_S551 | 39574 | 39909 | 336 | AATTGAGGCGGATGTACCAG | ATGATGGCAGCATTCCGTAT | InDel | 0.247 | 0.217 | 1.329 |
| S613_inID1 | Le_N7_S613 | 22967 | 23192 | 226 | CGCCTGATATTCTAGCCGAG | CTTGTGTGTAACGTGTCCCG | InDel | 0.390 | 0.351 | 1.641 |
| S635_inID1 | Le_N7_S635 | 71381 | 71574 | 194 | CCTGGTTGTCGCTCTTTCTC | CTGCAGCTTTTGTTCCCAAT | InDel | 0.427 | 0.336 | 1.745 |
| S636_inID1 | Le_N7_S636 | 91057 | 91346 | 290 | TCTCGGTATCATAGTCCCGC | TTGTCTTGAGCGCTGAGCTA | InDel | 0.439 | 0.343 | 1.784 |
| S640_inID1 | Le_N7_S640 | 30347 | 30694 | 348 | TAGAGAGCTCGCCATCCATT | ACTACGAATCATTGGCCGAC | InDel | 0.329 | 0.275 | 1.491 |
| S657_inID1 | Le_N7_S657 | 44849 | 45142 | 294 | AATCATGTAGGAGGCAACGG | GCTCCTGCTTGTGGATTCTC | InDel | 0.461 | 0.354 | 1.854 |
| S683_inID1 | Le_N7_S683 | 157151 | 157485 | 335 | AACCAGGGAAGGCCTGTTAT | TGCGTTTGAGACGAAACTTG | InDel | 0.494 | 0.377 | 1.977 |
| S701_inID1 | Le_N7_S701 | 6519 | 6773 | 255 | CGACGATTTCATGACACCAC | TGTGTACTGGGAGATGCTGC | InDel | 0.552 | 0.479 | 2.234 |

The scaffold number and the primer positions are derived from the genome sequence of shiitake strain L54A (Accession number: LOHM00000000). Gene diversity (*H*), polymorphism information content (PIC), effective number of alleles (*Ne*).
