# Supplementary material for: Association Mapping Reveals Genetic Loci Associated with Important Agronomic Traits in Lentinula edodes, Shiitake Mushroom
Source: Front Microbiol. 2017 Feb 17;8:237. doi: 10.3389/fmicb.2017.00237 (PMC5314409; doi:10.3389/fmicb.2017.00237)
Supplement: Supplementary file 3 [file Table3.doc]

**Supplementary Table S3. Analysis of variance for the nine fruiting body-related traits in 2013 and 2014.**

| Variables | Type III sum of square | | | Mean square | | | *F* value | | | *P* value | | |
| --- | --- | --- | --- | --- | --- | --- | --- | --- | --- | --- | --- | --- |
|  | Year | Genotype | Year×Genotype | Year | Genotype | Year×Genotype | Year | Genotype | Year×Genotype | Year | Genotype | Year×Genotype |
| df | 1 | 63* | 63 | 1 | 63 | 63 | 1 | 63 | 63 | 1 | 63 | 63 |
| PD | 4122.6 | 11873.79 | 4919.5 | 4122.6 | 188.47 | 78.09 | 165.59 | 7.57 | 3.14 | <0.001 | <0.001 | <0.001 |
| PT | 59.92 | 767.65 | 269.83 | 59.92 | 12.18 | 4.28 | 39.22 | 7.98 | 2.8 | <0.001 | <0.001 | <0.001 |
| PW | 637.52 | 5626.29 | 2328.64 | 637.52 | 89.31 | 36.96 | 45.76 | 6.41 | 2.65 | <0.001 | <0.001 | <0.001 |
| SD | 37.99 | 866.85 | 295.12 | 37.99 | 13.76 | 4.68 | 21.28 | 7.71 | 2.62 | <0.001 | <0.001 | <0.001 |
| SL | 594.9 | 12177.52 | 3498.12 | 594.9 | 193.29 | 55.53 | 37.88 | 12.31 | 3.54 | <0.001 | <0.001 | <0.001 |
| SW | 11.5 | 894.05 | 349.53 | 11.5 | 14.19 | 5.55 | 6.29 | 7.76 | 3.03 | 0.013 | <0.001 | <0.001 |
| NF | 5967.43 | 48135.17 | 12755.78 | 5967.43 | 764.05 | 202.47 | 167.6 | 21.46 | 5.69 | <0.001 | <0.001 | <0.001 |
| WF | 1049.67 | 8240.38 | 4221.6 | 1049.67 | 130.8 | 67.01 | 66.57 | 8.3 | 4.25 | <0.001 | <0.001 | <0.001 |
| Y | 40232.24 | 455243.7 | 265026 | 40232.24 | 7226.09 | 4206.76 | 32.95 | 5.92 | 3.45 | <0.001 | <0.001 | <0.001 |

Note: *During the two years’ cultivation, only 64 strains produced more than ten fruiting bodies both in 2013 and 2014, and used for two-way analysis of variance.
