# Supplementary material for: Association Mapping Reveals Genetic Loci Associated with Important Agronomic Traits in Lentinula edodes, Shiitake Mushroom
Source: Front Microbiol. 2017 Feb 17;8:237. doi: 10.3389/fmicb.2017.00237 (PMC5314409; doi:10.3389/fmicb.2017.00237)
Supplement: Supplementary file 4 [file Table4.doc]

**Supplementary Table S4.** **The *r^2^* of LD for pairwise loci screened on 89 shiitake cultivars.**

| Number of LD locus pairs | Frequency distribution of *r ^2^*(*p*< 0.001) | | | | | Mean of *r^2^* |
| --- | --- | --- | --- | --- | --- | --- |
|  | 0-0.2 | 0.2-0.4 | 0.4-0.6 | 0.6-0.8 | 0.8-1 |  |
| 19122 (43.50%) | 5744 | 8510 | 3381 | 1287 | 200 | 0.316 |
