# Supplementary material for: Association Mapping Reveals Genetic Loci Associated with Important Agronomic Traits in Lentinula edodes, Shiitake Mushroom
Source: Front Microbiol. 2017 Feb 17;8:237. doi: 10.3389/fmicb.2017.00237 (PMC5314409; doi:10.3389/fmicb.2017.00237)
Supplement: Supplementary file 5 [file Table5.doc]

**Supplementary Table S5. LD level averaged for map distance classes.**

| Genomic region (cM) | N* | *r^2^* |
| --- | --- | --- |
| 0-20 | 95 | 0.419 |
| 20-40 | 22 | 0.384 |
| 40-60 | 28 | 0.361 |
| >60 | 19 | 0.381 |
| intra-linkage group | 164 | 0.400 |
| inter-linkage group | 1188 | 0.327 |

* Number of marker pairs included in each class.
