# Supplementary material for: Association Mapping Reveals Genetic Loci Associated with Important Agronomic Traits in Lentinula edodes, Shiitake Mushroom
Source: Front Microbiol. 2017 Feb 17;8:237. doi: 10.3389/fmicb.2017.00237 (PMC5314409; doi:10.3389/fmicb.2017.00237)
Supplement: Supplementary file 6 [file Table6.doc]

**Supplementary Table S6.** **Twenty-four candidate genes containing or close to markers identified in both years or consistent with previous reports**.

| Marker | Scaffold | Trait | Gene | BLAST hit description |
| --- | --- | --- | --- | --- |
| S163_E1 | Le_N7_S163 | PW | aug_scv1_leest_g2887 | hypothetical protein GYMLUDRAFT_53118 [*Gymnopus luxurians* FD-317 M1] |
|  |  |  | aug_scv1_leest_g2888 | mismatched base pair and cruciform DNA recognition protein [*Coniophora puteana* RWD-64-598 SS2] |
|  |  |  | aug_scv1_leest_g2889 | Cytochrome P450 |
| S48_ID1 | Le_N7_S48 | WF | aug_scv1_leest_g570 | Aldo/keto reductase [*Laetiporus sulphureus* 93-53] |
|  |  |  | aug_scv1_leest_g571 | Aldo/keto reductase [*Gloeophyllum trabeum* ATCC 11539] |
|  |  |  | aug_scv1_leest_g572 | glycoside hydrolase family 27 protein [*Gymnopus luxurians* FD-317 M1] |
| S560_ID1 | Le_N7_S560 | WF | aug_scv1_leest_g10783 | sucrose cleavage family protein [*Moniliophthora roreri* MCA 2997] |
|  |  | PW | aug_scv1_leest_g10784 | predicted protein [*Laccaria bicolor* S238N-H82] |
|  |  | PD | aug_scv1_leest_g10785 | serine threonine kinase |
| S255_ID1 | Le_N7_S255 | PW | aug_scv1_leest_g5310 | P-loop containing nucleoside triphosphate hydrolase protein |
|  |  | WF | aug_scv1_leest_g5311 | hypothetical protein GYMLUDRAFT_35665 [*Gymnopus luxurians* FD-317 M1] |
| S346_ID1 | Le_N7_S346 | SW | aug_scv1_leest_g7116 | AAA-domain-containing protein [*Fibulorhizoctonia sp.* CBS 109695] |
|  |  |  | aug_scv1_leest_g7117 | ribosomal protein l30 |
| S278_ID4 | Le_N7_S278 | PD | aug_scv1_leest_g5758 | predicted protein [*Laccaria bicolor* S238N-H82] |
|  |  |  | aug_scv1_leest_g5759 | hypothetical protein SCHCODRAFT_255575 [*Schizophyllum commune* H4-8] |
| S328_ID5 | Le_N7_S328 | PW | aug_scv1_leest_g6792 | cyclin-dependent protein kinase inhibitor [*Coprinopsis cinerea* okayama7#130] |
|  |  |  | aug_scv1_leest_g6793 | hypothetical protein GYMLUDRAFT_158815 [*Gymnopus luxurians* FD-317 M1] |
|  |  |  | aug_scv1_leest_g6794 | single-stranded DNA-binding protein [*Moniliophthora roreri* MCA 2997] |
| S704_inID1 | Le_N7_S704 | PW | aug_scv1_leest_g12045 |  |
|  |  | PD | aug_scv1_leest_g12046 |  |
| S278_ID10 | Le_N7_S278 | PD PW | aug_scv1_leest_g5720 | alkaline protein involved in sphingolipid metabolism [*Moniliophthora roreri* MCA 2997] |
| S127_ID1 | Le_N7_S121 | PW | aug_scv1_leest_g2020 | ring finger domain protein [*Moniliophthora roreri* MCA 2997] |
|  |  |  | aug_scv1_leest_g2021 | proline-rich family protein [*Moniliophthora roreri* MCA 2997] |
|  |  |  | aug_scv1_leest_g2022 | predicted protein [*Laccaria bicolor* S238N-H82] |
