# Supplementary material for: Association Mapping Reveals Genetic Loci Associated with Important Agronomic Traits in Lentinula edodes, Shiitake Mushroom
Source: Front Microbiol. 2017 Feb 17;8:237. doi: 10.3389/fmicb.2017.00237 (PMC5314409; doi:10.3389/fmicb.2017.00237)
Supplement: Supplementary file 7 [file Image1.pdf]

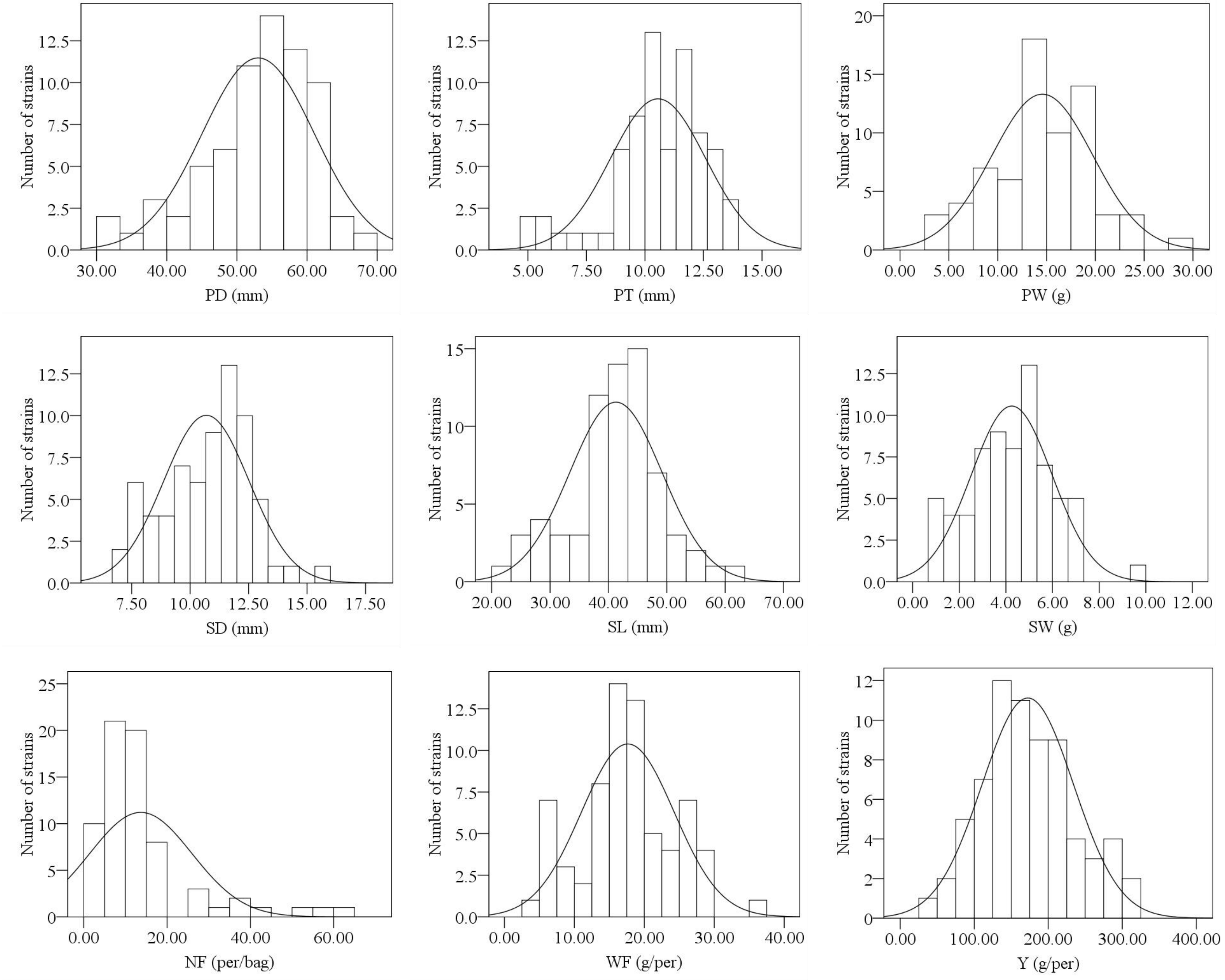

Supplementary Figure S1. Histograms showing frequency distribution of nine agronomic traits in 2014. The y-axis denotes the number of strains, whereas the x-axis indicates value range of traits.
