# Supplementary material for: Association Mapping Reveals Genetic Loci Associated with Important Agronomic Traits in Lentinula edodes, Shiitake Mushroom
Source: Front Microbiol. 2017 Feb 17;8:237. doi: 10.3389/fmicb.2017.00237 (PMC5314409; doi:10.3389/fmicb.2017.00237)
Supplement: Supplementary file 8 [file Image2.PDF]

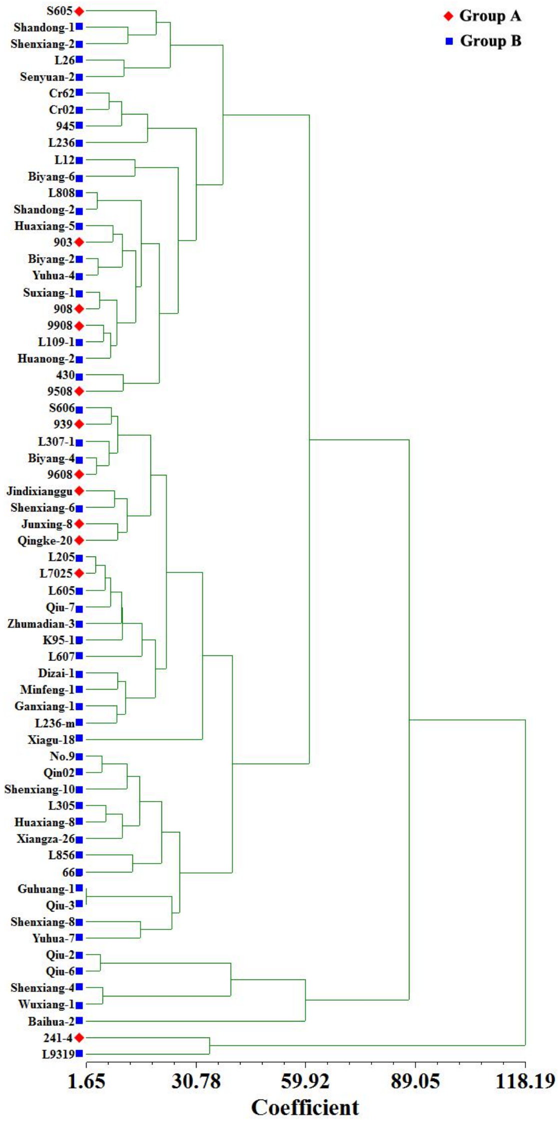

Supplementary Figure S2 UPGMA dendrogram based on the phenotypic data of 64 strains. ◆, strains in Group A of NJ tree based on SSR and InDel markers; ■, strains in Group B.
