# Supplementary material for: Association Mapping Reveals Genetic Loci Associated with Important Agronomic Traits in Lentinula edodes, Shiitake Mushroom
Source: Front Microbiol. 2017 Feb 17;8:237. doi: 10.3389/fmicb.2017.00237 (PMC5314409; doi:10.3389/fmicb.2017.00237)
Supplement: Supplementary file 9 [file Image3.PDF]

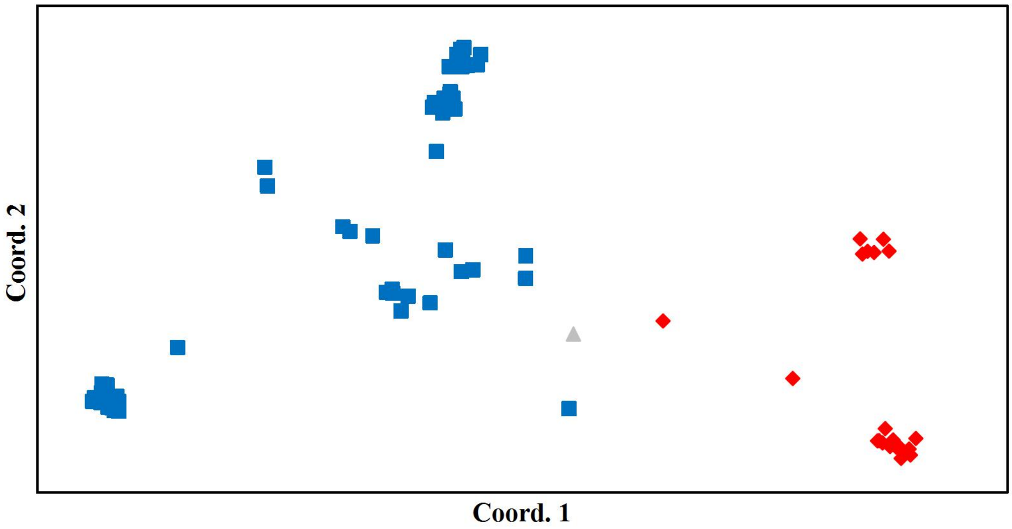

Supplementary Figure S3 Principal coordinate analysis of 89 *Lentinula edodes* cultivars. Strains from the different groups defined in the NJ tree are marked in different colors symbols: ◆， Group A；■, Group B； ▲， Xiangjiu , an outlier strain.
