# Supplementary material for: Association Mapping Reveals Genetic Loci Associated with Important Agronomic Traits in Lentinula edodes, Shiitake Mushroom
Source: Front Microbiol. 2017 Feb 17;8:237. doi: 10.3389/fmicb.2017.00237 (PMC5314409; doi:10.3389/fmicb.2017.00237)
Supplement: Supplementary file 10 [file Image4.PDF]

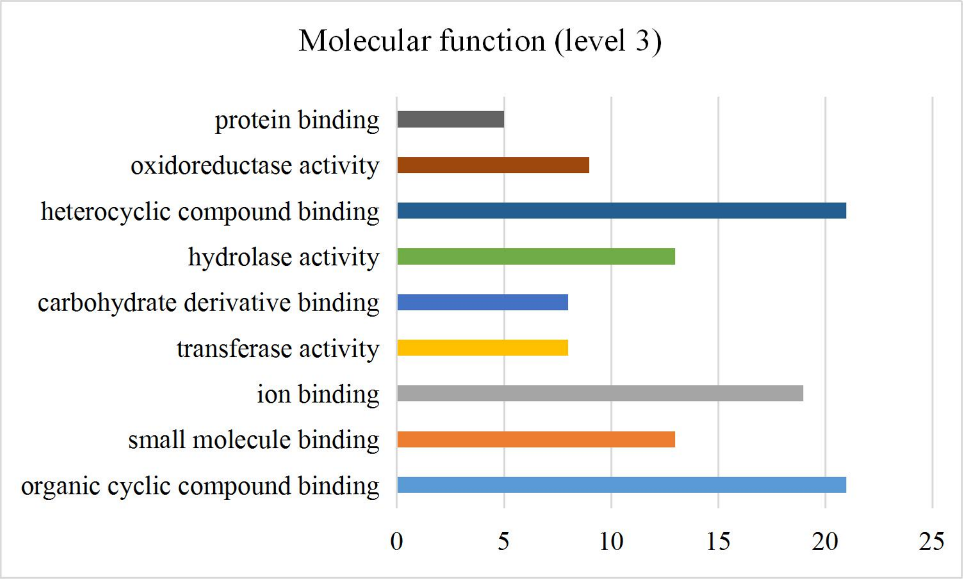

Supplementary Figure S4 Gene ontology (GO) annotation of 97 candidate genes associated with agronomic traits in *Lentinula edodes* using Blast2GO.
