# Supplementary material for: Association Mapping Reveals Genetic Loci Associated with Important Agronomic Traits in Lentinula edodes, Shiitake Mushroom
Source: Front Microbiol. 2017 Feb 17;8:237. doi: 10.3389/fmicb.2017.00237 (PMC5314409; doi:10.3389/fmicb.2017.00237)
Supplement: Supplementary file 11 [file Image5.pdf]

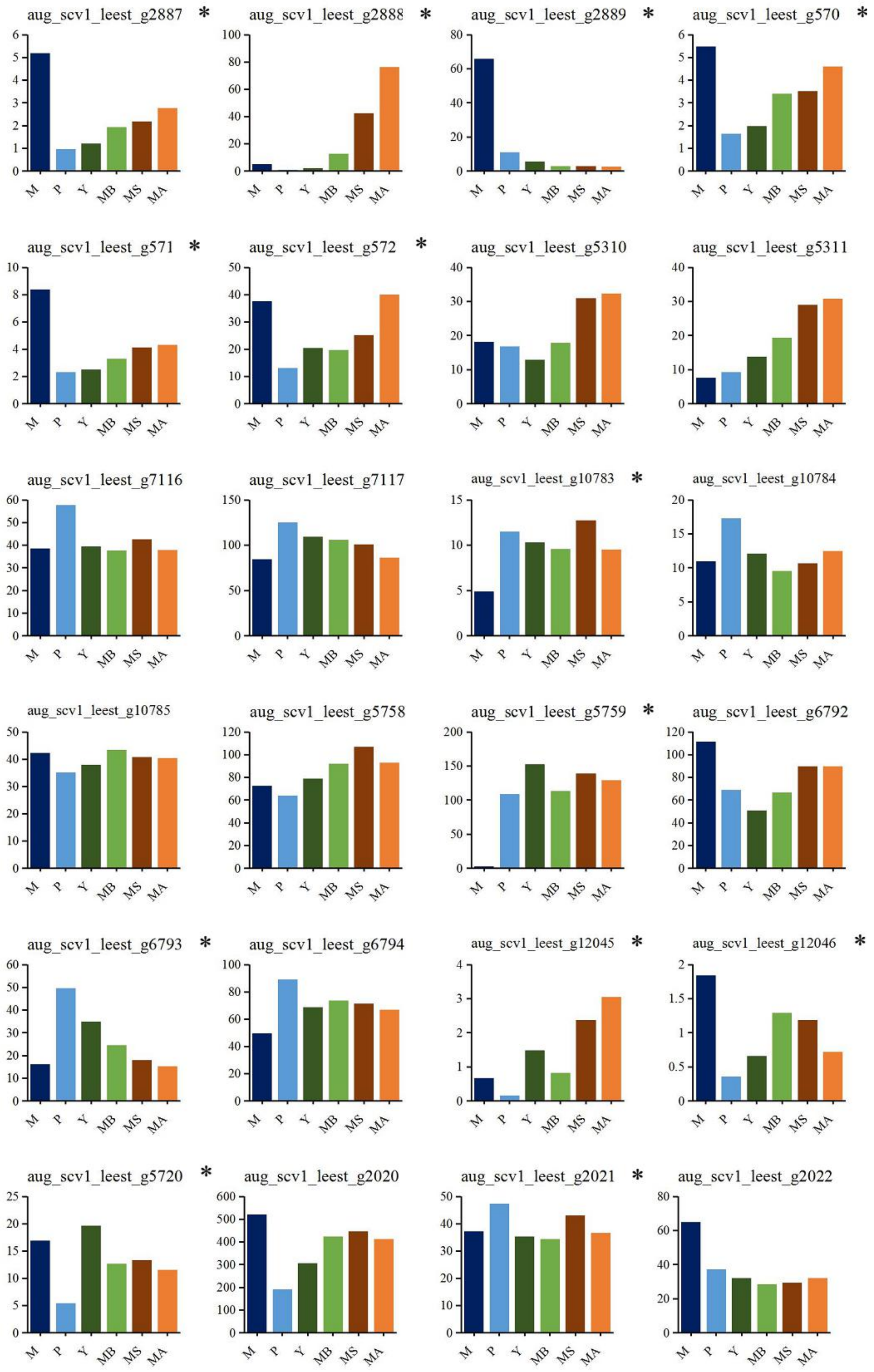

Supplementary Figure S5. Expression levels of 24 genes containing or close to

trait-associated markers identified previously or detected in both years in shiitake strain L54. Data here are from our unpublished RNA-seq data. Gene expression levels were normalized by RPKM (reads per kilo base per million mapped reads). M: Mycelium incubated in 12 hours light/12 hours dark; P: Primordium; Y: Young fruiting body; MB: Mature fruiting body before sporulation; MS: Mature fruiting body during sporulation; MA: Mature fruiting body after sporulation. * Genes with a >2-fold expression change between M and P stages.
